# Research on the equity of health manpower resource allocation in the Yangtze River Delta region

**DOI:** 10.3389/fpubh.2025.1650147

**Published:** 2025-10-14

**Authors:** Qinglian Li, Siyuan Zheng, Zhengwei Jing, Dingwan Chen, Kun Chen, Yingjun Li

**Affiliations:** ^1^Department of Epidemiology and Health Statistics, School of Public Health, Hangzhou Medical College, Hangzhou, China; ^2^Department of Social Medicine and Health Services Management, School of Public Health, Hangzhou Medical College, Hangzhou, China; ^3^Department of Zhejiang, University School of Public Health, Hangzhou, China

**Keywords:** Yangtze River Delta region, health technicians, the Gini coefficient, the Theil index, health resource agglomeration degree

## Abstract

**Background:**

To analyze the equity of the current allocation of health human resources using statistical data on health resources in the Yangtze River Delta region.

**Methods:**

The Gini coefficient quantifies the level of distributional equality, the Theil index assesses the source of inequality, and the health resource agglomeration degree (HRAD) measures the accessibility of health resources, combining the three methods to evaluate the equity of the current allocation of health human resources in the Yangtze River Delta region. Furthermore, trend analysis of fairness indicators was conducted using regression models.

**Results:**

Human resources for health in the Yangtze River Delta region have been increasing between 2014 and 2022. The Gini coefficient and Theil index in the Yangtze River Delta region are more equitable in terms of the distribution of healthcare resources based on population and gross domestic product (GDP) rather than geographical region. In Anhui Province, HRAD and HRAD/PAD (population agglomeration degree) were both less than 1. In Zhejiang Province, HRAD for health technicians and registered nurses was less than 1.

**Conclusion:**

Human resources for health and healthcare ratios in the Yangtze River Delta region have continued to grow. However, the equity of health resources allocated based on population and economic factors is superior to that allocated based on geographical factors, and the equity of health resource concentration remains to be improved. To address this equity issue, it is necessary to comprehensively consider various factors such as population, geography, and GDP, and formulate corresponding measures accordingly.

## 1 Background

The World Health Organization (WHO) health policy explicitly urges governments to take effective measures to reduce health disparities among different populations, especially for vulnerable groups, and to ensure equitable access to health services (1). In view of this global consensus, China has established a national strategy for the balanced distribution of high-quality healthcare resources through the ‘Healthy China 2030’ initiative and the ‘14th Five-Year National Health Plan’ (2).

Compared to western regions, the Beijing-Tianjin-Hebei region, and the Pearl River Delta, the Yangtze River Delta region faces unique challenges: within this highly developed economic region, significant development disparities and geographical barriers have led to an uneven distribution of healthcare human resources (3, 4). Specifically, as the Yangtze River Delta experiences rapid socio-economic development, residents’ healthcare needs continue to rise. However, significant disparities exist within the region: core areas such as Shanghai, southern Jiangsu, and northern Zhejiang are highly developed, while some peripheral regions are constrained by terrain and transportation limitations (5). This dual economic-geographical influence has led to an imbalance in the allocation of healthcare human resources, creating a major challenge in coordinating population health needs with healthcare service supply. This not only hinders the formation of a fair, convenient, and systematic healthcare service system but also limits the comprehensive implementation of the ‘people-centred healthcare’ strategy (6, 7).

Therefore, in order to implement national strategic goals and take the lead in achieving health modernization in the region, promoting the balanced development of health technicians is a fundamental guarantee for ensuring health equity. Based on the current state of health resource development, providing targeted recommendations for the allocation of health resources in the Yangtze River Delta is crucial for promoting the sustainable development of healthcare and optimizing resource allocation in the region.

This study uses the Gini coefficient (G), the Health Resource Agglomeration Degree (HRAD), and the Theil index (T) to comprehensively assess the fairness of public health human resource allocation in the Yangtze River Delta from 2014 to 2022 (8–10). The design of this indicator combination is based on the principle of multidimensional complementarity: the Gini coefficient excels at quantifying the overall inequality of resource allocation and effectively identifying global imbalances, but it has limitations in analysing spatial structures and the sources of differences; HRAD integrates population density and geographical area parameters to precisely capture spatial accessibility bottlenecks in health services, addressing the Gini coefficient’s blind spots in the geographical dimension, and the Theil index, through its decomposable characteristics, precisely identifies the contribution rates of differences between and within regions, revealing the underlying structural causes of inequality (11). The three indicators cross-validate each other from multiple dimensions, breaking through the cognitive boundaries of a single indicator.

## 2 Methods

### 2.1 Data source

In this research, ‘health workers’ refers to employees working in hospitals, primary health care institutions, professional public health organizations, and other health care institutions, using the classification standards of the China Health Statistics Yearbook: health technicians include practising (assistant) physicians, registered nurses, pharmacists, and other clinical service positions; other health technicians refer to professional and technical positions other than health technicians, village doctors, labourers, and management personnel. The distribution of health personnel and the relevant statistics per 10,000 people in the Yangtze River Delta region were primarily obtained from the China Statistical Yearbook (2015–2023) on the official website of the National Bureau of Statistics. Additionally, data on the gross domestic product, healthcare personnel, permanent population, and geographical areas of prefecture-level cities in the Yangtze River Delta region are sourced from the Statistical Yearbooks and Statistical Bulletins on National Economic and Social Development (2015–2023) of Jiangsu Province, Zhejiang Province, Shanghai Municipality, and Anhui Province.

### 2.2 Measuring tools

#### 2.2.1 Gini coefficients

The G is a statistical indicator calculated based on the Lorenz curve, reflecting the fairness of social income distribution (10, 12). Currently, the G is widely used to assess the fairness of medical resource allocation. The G takes values ranging from 0 to 1, with 0.4 as the warning line. Generally, 0 ~ 0.2 indicates high fairness, 0.2 ~ 0.3 indicates comparative fairness, 0.3 ~ 0.4 indicates relative fairness, and greater than 0.4 indicates unfairness (10, 13). In this study, the G was used to analyze the equity of health human resource allocation among regions in the Yangtze River Delta based on population, GDP and geographical area. The formula for calculating G is as shown in Equation 1:


(1) $$G=1-\sum_{i=1}^{n-1}(X_{i+1}-X_{i})(Y_{i+1}-Y_{i})$$

Where *G* is the Gini coefficient, *n* represents the number of geographic regions, and *i* takes values from 1 to *n*. The provinces are sorted in ascending order by the number of health personnel per 10,000 population (100 million yuan or per square kilometer). *Y_i_* denotes the cumulative proportion of health human resources in the *i*th region, while *X_i_* represents the cumulative proportion of the population (GDP or geographic area) in the *i*th region.

#### 2.2.2 Theil index

The Theil index, derived from the concept of entropy in information theory, is used to measure the equity of health resource allocation in a region (10, 13). The Theil index has good decomposition capabilities and is a commonly used method for assessing the balance of resource allocation and analysing the causes of uneven health resource allocation in the Yangtze River Delta region. It can reflect the magnitude of differences within and between regions, and the calculation of contribution rates can intuitively show the extent to which regions within and between regions contribute to overall differences. The value of the Theil index ranges from 0 to 1, with smaller values indicating greater fairness (14, 15). Its calculation formula is as the following Equations 2–5.


(2) $$T=\sum_{i=1}^{n}F_{i}\log\frac{F_{i}}{P_{i}}$$

Where *T* denotes the Theil index, *P_i_* is the percentage of the resident population (GDP or geographic area) of each prefecture-level city to the total population (GDP or geographic area) of the four provinces in the Yangtze River Delta region (Jiangsu, Zhejiang, Shanghai, and Anhui); *F_i_* is the percentage of the number of human resources for health in each prefecture-level city to the number of human resources for health in the four provinces in the Yangtze River Delta region; *i* denotes the province, and n denotes the total number of provinces, and the method of calculating the decomposition of the Theil index:


(3) $$T=T_{\text{inter}}+T_{\text{intra}}$$

(4) $$T_{\text{inter}}=\sum_{k=1}^{m}F_{k}\ln\frac{F_{k}}{P_{k}}$$

(5) $$T_{\text{intra}}=\sum_{k=1}^{m}F_{k}T_{k}$$

*T_total_* denotes the total Theil index, *T_inter_* refers to the differences in resource allocation between provinces in the Yangtze River Delta region; *T_intra_* denotes the differences in resource allocation within provinces in the Yangtze River Delta region; *P_k_* is the percentage of the resident population (GDP or geographic area) of each province to the total population (GDP or geographic area) of the Yangtze River Delta region, *F_k_* is the number of human resources for health in each province to the number of human resources for health in the Yangtze River Delta region, and *T_k_* denotes each regional Theil index, *k* denotes prefecture-level cities, and *m* denotes the total number of prefecture-level cities.

The intra-group contribution rate is calculated as *T_intra_/T_total_* × 100%, while the inter-group contribution rate is calculated as *T_inter_/T_total_* × 100%.

#### 2.2.3 Health resource agglomeration degree

Agglomeration is an indicator of the degree to which health resources are clustered in a region relative to a larger regional scale, and is categorized into health resource agglomeration degree (HRAD) and population agglomeration degree (PAD) (16). HRAD is used to measure the extent to which human resources for health are clustered in a given region and how this varies across regions; HRAD/PAD is used to measure whether regional health resources meet the needs of the local population (17). The degree of agglomeration is calculated as shown in Equations 6, 7.


(6) $${\text{HRAD}}_{i}=\frac{(\frac{{\mathrm{HR}}_{i}}{{\mathrm{HR}}_{n}})\times 100\%}{(\frac{A_{i}}{A_{n}})\times 100\%}=\frac{\frac{{\mathrm{HR}}_{i}}{A_{i}}}{\frac{{\mathrm{HR}}_{n}}{A_{n}}}$$

(7) $${\mathrm{PAD}}_{i}=\frac{(P_{i}/P_{n})\times 100\%}{(A_{i}/A_{n})\times 100\%}=\frac{P_{i}/A_{i}}{P_{n}/A_{n}}$$

*HRAD_i_* denotes the agglomeration of human resources for health in area *i*, where *HR_i_* is the number of health personnel in area *i*, *A_i_* is the land area of area *i*, *A_n_* is the total land area of the country, and *HR_n_* is the total number of health personnel in the country. *PAD_i_* denotes the population density of area *i*, where *P_i_* is the number of people in area *i*, and *P_n_* is the total population of the country.

When *HRAD_i_ = 1*, it means that the human resources in the region are absolutely fair by geographic allocation; when *HRAD_i_ > 1*, it means that the fairness of human resources in the region is better by geographic allocation; on the contrary, *HRAD_i_ < 1* means that the fairness is worse. When *HRAD* and *PAD* are combined to evaluate the equity of health resources, the evaluation criteria are as follows: when *HRAD_i_/PAD_i_ = 1*, it indicates that the human resources in the region are allocated absolutely equitably according to the population, and the accessibility of health resources is better; when the ratio of the two is *>1*, it indicates that the human resources in the region are allocated equitably according to the population is better; and vice versa, *<1* indicates that the poor equity (18).

#### 2.2.4 Statistical analysis

To quantify the temporal trends and statistical significance of the aforementioned fairness indicators, this research constructed univariate linear regression models with year as the independent variable and each fairness indicator as the dependent variable. Details of the linear regression results are provided in Supplementary Tables 23–28. The slope of each regression model was tested using a *t*-test, and its 95% confidence interval was calculated. All data analyses were performed using R (version 4.4.1).

## 3 Results

### 3.1 Situation of the scale of health technology personnel in the Yangtze River Delta region

Numbers of all types of health technicians and health care ratios show an upward trend from 2014 to 2022. In terms of human resources for health, the growth rate of health technicians, licensed (assistant) physicians, registered nurses, pharmacists and other health technicians is 60.58, 63.08, 75.33, 34.67, 68.82%, respectively, the fastest for registered nurses and the slowest for pharmacists.

Besides, from 2014 to 2022, the medical staff ratio in the Yangtze River Delta region will generally increase. However, by 2022, the medical staff ratio in Zhejiang, Jiangsu, and Anhui provinces will still be less than 1:2, with only Shanghai reaching a ratio of more than 1:2. The specific numbers are shown in Table 1.

**Table 1:** Number of human resources for health (unit: 10,000 persons) and healthcare ratio* in the Yangtze River Delta region, 2014–2022.

Province	Year	Health technician	licensed (assistant) physicians	Registered nurse	Pharmacists	Other health technicians	Healthcare ratio
Jiangsu	2014	45.85	17.86	18.88	2.55	2	1:1.06
	2015	48.7	18.92	20.4	2.78	2.08	1:1.08
	2016	51.7	20.46	22.12	2.78	2.52	1:1.08
	2017	54.77	21.71	23.69	2.88	2.95	1:1.09
	2018	59.01	23.33	26.04	3.02	3.19	1:1.12
	2019	63.33	25.47	27.98	3.15	3.47	1:1.10
	2020	66.55	26.78	29.42	3.26	3.72	1:1.10
	2021	69.18	27.27	30.87	3.43	4.32	1:1.13
	2022	71.37	27.92	31.83	3.57	4.21	1:1.14
Zhejiang	2014	37.59	14.57	14.51	2.45	1.81	1:1.00
	2015	40.56	15.81	15.99	2.58	1.88	1:1.01
	2016	43.26	16.82	17.45	2.69	2.04	1:1.04
	2017	45.97	17.87	18.77	2.79	2.22	1:1.05
	2018	48.62	19.08	20.15	2.9	2.48	1:1.06
	2019	52.02	20.55	21.98	3.03	2.67	1:1.07
	2020	54.8	21.77	23.31	3.14	2.74	1:1.07
	2021	57.91	23.27	25.03	3.25	2.87	1:1.08
	2022	61.28	24.66	26.7	3.3	2.91	1:1.08
Shanghai	2014	16.41	6.12	7.19	0.92	0.99	1:1.17
	2015	17.01	6.3	7.54	0.95	1.02	1:1.20
	2016	17.82	6.54	7.94	0.98	1.07	1:1.21
	2017	18.69	6.79	8.39	1	1.15	1:1.24
	2018	19.56	7.16	8.8	1.02	1.16	1:1.23
	2019	20.45	7.47	9.29	1.04	1.17	1:1.24
	2020	21.44	7.84	9.72	1.07	1.17	1:1.24
	2021	22.9	8.41	10.39	1.12	1.28	1:1.24
	2022	23.6	8.6	10.64	1.13	1.32	1:1.24
Anhui	2014	26.8	10.37	11.5	1.29	1.39	1:1.11
	2015	28.08	10.78	11.93	1.35	1.44	1:1.11
	2016	29.37	11.27	12.64	1.39	1.47	1:1.12
	2017	31.35	12.08	13.81	1.45	1.51	1:1.14
	2018	33.36	12.68	14.97	1.5	1.65	1:1.18
	2019	36.12	13.84	16.34	1.57	1.8	1:1.18
	2020	41.21	16.42	18.82	1.67	1.88	1:1.15
	2021	43.51	17.26	20.1	1.71	2.1	1:1.16
	2022	47.12	18.6	22.14	1.71	2.01	1:1.19

*All data on healthcare personnel are sourced from the China Statistical Yearbook 2015–2022.

### 3.2 Variation in health technology personnel per 10,000 persons in the Yangtze River Delta region

**Figure 1:**
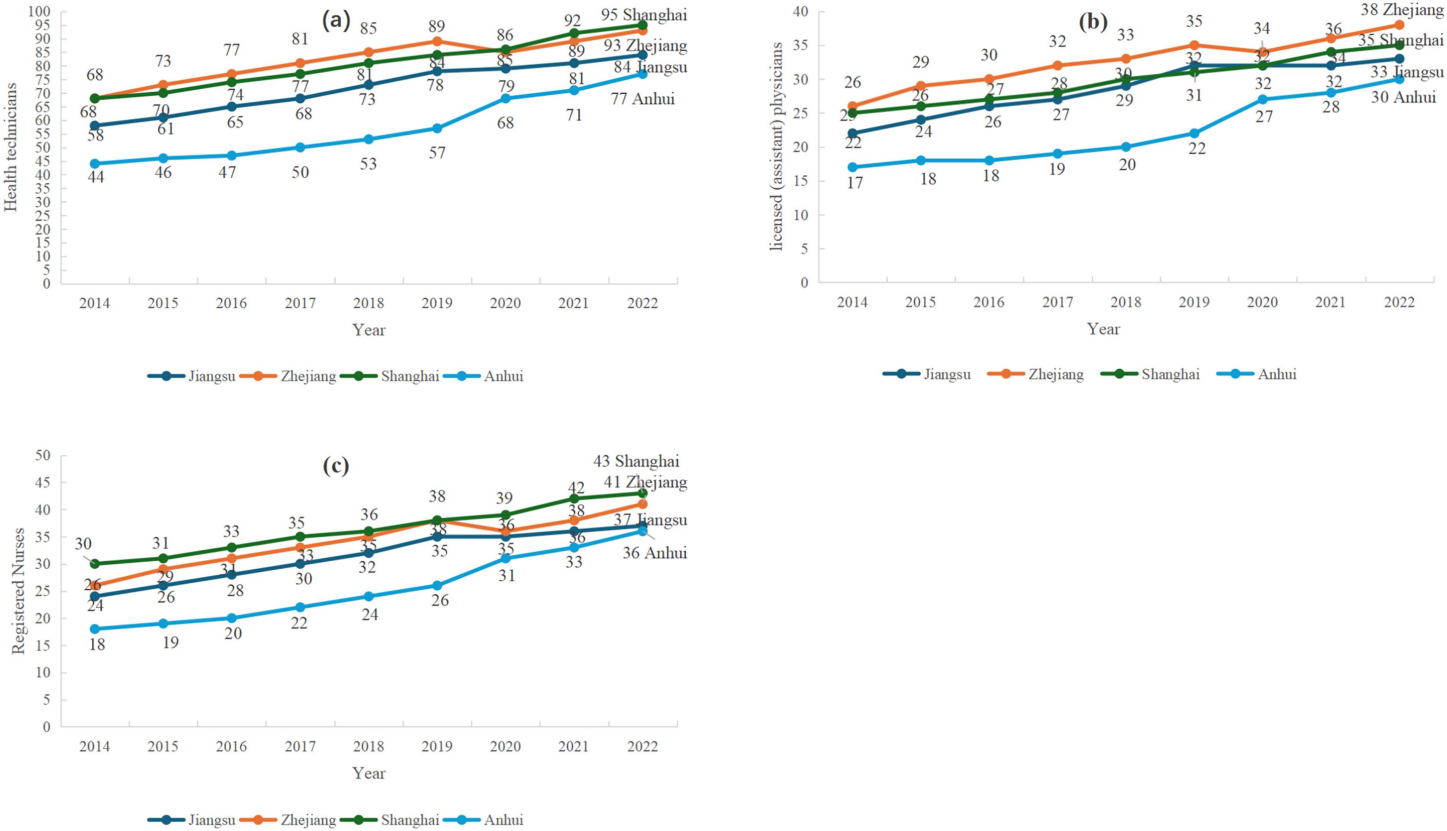
Trends in the Number of Three Categories of Healthcare Personnel per 10,000 Population in the Yangtze River Delta region from 2014 to 2022. **(a)** Health Technicians, **(b)** Registered nurses, **(c)** Licensed (Assistant) Physicians. *Provincial Health Statistical Yearbooks (Jiangsu, Zhejiang, Shanghai, Anhui) 2015–2023.

The Yangtze River Delta data on health technicians, licensed (assistant) physicians and registered nurses per 10,000 people from 2014 to 2022 are shown in Figure 1. Overall, the number of health technicians, licensed (assistant) physicians and registered nurses per 10,000 people from 2014 to 2022 in Jiangsu, Zhejiang, Shanghai and Anhui provinces are all increasing. In Anhui Province, there is a noticeable gap between the number of health technicians, licensed (assistant) physicians, and registered nurses per 10,000 people compared to those in Jiangsu, Zhejiang, and Shanghai; however, this gap is gradually narrowing.

### 3.3 The Gini coefficient-based equity analysis

According to the Gini coefficient fairness analysis by population configuration, the Gini coefficients of health technicians, licensed (assistant) physicians, and registered physicians in Jiangsu, Zhejiang, and Anhui provinces are all less than 0.2, indicating a highly equitable state, reflecting the high match between the permanent resident population and medical resources; the Gini coefficients of the three categories of human resources for health in Shanghai city are located in the range of 0.3–0.4, which is in a relatively fair state. This may be attributed to Shanghai’s status as a developed city, where population mobility significantly influences the distribution of healthcare human resources.

According to the Gini coefficient fairness analysis of geographic area configuration, the Gini coefficient of the three types of health human resources in Jiangsu Province and Anhui Province is located between 0.2–0.4, in a relatively fair and relatively fair state; the Gini coefficient of the three types of health human resources in Zhejiang Province is located between 0.3–0.4, in a relatively fair state. This may be due to geographical constraints within these regions. Both transportation and healthcare services have an impact on this distribution. However, the Gini coefficients for the three types of healthcare human resources in Shanghai exceed 0.5, indicating an unfair state. This may be due to Shanghai’s healthcare distribution potentially forming a “concentric circle” pattern, where resources are more abundant closer to the center.

Based on GDP, the Gini coefficient analysis shows that the G for the three categories of healthcare human resources in Zhejiang and Jiangsu are all below 0.2; the G for the three categories in Shanghai are in the range of 0.3–0.4; Anhui shows a differentiation—the G for healthcare technicians and licensed physicians fluctuates around 0.2, while the G for nurses remains consistently below 0.2. This reflects relatively lower economic pressure, but none of the four provinces have achieved a highly equitable state, indicating that underdeveloped regions within these provinces still require improvements in the allocation of healthcare resources. The fairness status of the Gini coefficient for Zhejiang is shown in Table 2, and the Gini coefficients for Jiangsu, Shanghai, and Anhui are shown in Supplementary Tables 1–9, respectively. The trend charts for the Gini coefficients are detailed in Supplementary Figures 2–5.

**Table 2:** The Gini coefficients for the three categories of human resources for health in Zhejiang, 2014–2022.

Province	Year	Population-based allocation			Geographic area-based allocation			GDP-based allocation		
		Health technicians	licensed (assistant) physicians	Registered nurses	Health technicians	licensed (assistant) physicians	Registered nurses	Health technicians	licensed (assistant) physicians	Registered nurses
Zhejiang	2014	0.142	0.124	0.158	0.263	0.252	0.273	0.177	0.130	0.111
	2015	0.135	0.119	0.146	0.259	0.247	0.268	0.122	0.132	0.117
	2016	0.134	0.118	0.142	0.253	0.242	0.262	0.121	0.136	0.117
	2017	0.137	0.123	0.144	0.259	0.248	0.266	0.129	0.143	0.127
	2018	0.138	0.123	0.143	0.260	0.252	0.267	0.124	0.138	0.123
	2019	0.137	0.127	0.143	0.262	0.255	0.264	0.122	0.136	0.123
	2020	0.143	0.130	0.150	0.270	0.268	0.276	0.116	0.120	0.114
	2021	0.142	0.132	0.148	0.273	0.274	0.277	0.123	0.125	0.124
	2022	0.139	0.129	0.143	0.275	0.278	0.278	0.127	0.128	0.128

*Prefecture-level city GDP (billion yuan), permanent population (10,000 people), land area (km^2^), and health personnel classification data are sourced from the health statistics data of the Zhejiang Health Commission and the 2015–2023 Statistical Yearbooks of Yangtze River Delta.

### 3.4 The Theil index based fairness analysis

According to the equity analysis of the Theil index of population allocation, the Theil index of health technicians, licensed (assistant) physicians, and registered nurses in the Yangtze River Delta region is less than 0.04. The contribution rate of intra-regional differences has consistently exceeded 59%, with internal regional differences contributing significantly more to the overall index than inter-regional differences.

Equity analysis of the Theil index of geographic configuration shows that the total Theil index of the three types of health human resources from 2014 to 2022 is mainly concentrated in the range of 0.177–0. 226, with little overall fluctuation, but the Theil index of the geographic configuration is larger than the Theil index of the population configuration, and the smallest Theil index of licensed (assistant) physicians in 2020, at 0.177, with the best configuration equity; the Theil index for registered nurses in 2018 is the largest, 0.226, with the worst configuration equity. The contribution rate of intra-regional differences ranged from 54.21 to 63.76%.

The Theil index analysis based on GDP allocation (2014–2022) shows that the Theil index range for the three categories of healthcare human resources is 0.028 to 0.040, with the contribution rate of regional differences stabilising above 62.92%. Among these, the Theil index for medical technical personnel was the lowest in 2019 (0.028), indicating the highest fairness in distribution. Further analysis indicates that geographical distribution differences in the Theil index are most significant in the Yangtze River Delta region, followed by GDP distribution differences, while population distribution differences are the smallest. As shown in Table 3.

**Table 3:** The Theil Index for the three categories of human resources for health, 2014–2022.

Theil Index	2014	2015	2016	2017	2018	2019	2020	2021	2022
Population-based allocation									
Health technicians									
Overall Theil Index	0.030	0.030	0.029	0.030	0.032	0.031	0.024	0.026	0.025
Contribution of interregional variations%	35.11%	38.10%	40.12%	40.22%	37.60%	37.17%	26.52%	24.14%	21.55%
Contribution to intraregional variations%	64.89%	61.90%	59.88%	59.78%	62.40%	62.83%	73.48%	75.86%	78.55%
Licensed (assistant) physicians									
Overall Theil Index	0.027	0.028	0.026	0.027	0.029	0.029	0.022	0.024	0.025
Contribution of interregional variations%	33.50%	37.01%	40.82%	40.52%	39.01%	39.36%	29.56%	29.78%	30.30%
Contribution to intraregional variations%	66.50%	62.99%	59.18%	59.48%	60.99%	60.64%	70.44%	70.22%	69.70%
Registered nurses									
Overall Theil Index	0.035	0.034	0.032	0.032	0.035	0.034	0.028	0.029	0.029
Contribution of interregional variations%	21.35%	24.73%	26.99%	26.85%	24.92%	25.40%	18.61%	18.62%	18.06%
Contribution to intraregional variations%	78.64%	75.27%	73.01%	73.15%	75.08%	74.60%	81.38%	81.38%	81.94%
Geographic area-based allocation									
Health technicians									
Overall Theil Index	0.191	0.189	0.194	0.196	0.207	0.202	0.195	0.199	0.194
Contribution of interregional variations%	45.79%	45.46%	45.47%	44.93%	43.91%	43.00%	41.73%	40.63%	39.28%
Contribution to intraregional variations%	54.21%	54.54%	54.53%	55.07%	56.09%	57.00%	58.27%	59.37%	60.72%
Licensed (assistant) physicians									
Overall Theil Index	0.183	0.180	0.182	0.182	0.194	0.187	0.177	0.182	0.179
Contribution of interregional variations%	44.02%	44.35%	44.60%	43.55%	42.94%	42.04%	39.85%	38.75%	37.67%
Contribution to intraregional variations%	55.98%	55.65%	55.40%	56.45%	57.06%	57.96%	60.15%	61.25%	62.33%
Registered nurses									
Overall Theil Index	0.218	0.212	0.215	0.215	0.226	0.217	0.208	0.208	0.201
Contribution of interregional variations%	41.71%	41.50%	41.68%	41.05%	40.37%	39.60%	38.53%	37.72%	36.24%
Contribution to intraregional variations%	58.29%	58.50%	58.32%	58.95%	59.63%	60.40%	61.47%	62.28%	63.76%
GDP-based allocation									
Health technicians									
Overall Theil Index	0.033	0.034	0.033	0.033	0.034	0.028	0.029	0.030	0.031
Contribution of interregional variations%	19.43%	21.01%	21.59%	20.90%	16.88%	17.99%	20.80%	20.76%	23.58%
Contribution to intraregional variations%	80.57%	78.99%	78.41%	79.10%	83.12%	82.01%	79.20%	79.24%	76.42%
Licensed (assistant) physicians									
Overall Theil Index	0.036	0.038	0.038	0.039	0.038	0.031	0.034	0.036	0.037
Contribution of interregional variations%	33.81%	33.33%	32.54%	33.12%	27.90%	27.08%	33.28%	34.51%	37.08%
Contribution to intraregional variations%	66.19%	66.67%	67.46%	66.88%	72.10%	72.92%	66.72%	65.49%	62.92%
Registered nurses									
Overall Theil Index	0.035	0.036	0.037	0.038	0.040	0.032	0.035	0.037	0.040
Contribution of interregional variations%	32.12%	33.06%	31.79%	32.39%	25.94%	24.44%	29.84%	30.22%	34.88%
Contribution to intraregional variations%	67.82%	66.94%	68.21%	67.61%	74.06%	75.56%	70.16%	69.78%	65.12%

*Prefecture-level city GDP (billion yuan), permanent population (10,000 people), land area (km^2^), and health personnel classification data are sourced from the health statistics data of the Zhejiang Health Commission and the 2015–2023 Statistical Yearbooks of Yangtze River Delta.

Research data indicates that intra-regional variations significantly exceed inter-regional variations across the three dimensions. This pattern likely stems from the potent ‘siphon effect’ exerted by economic hubs such as Shanghai, Hangzhou, and Nanjing, which attract high-quality medical resources through their generous remuneration packages and career opportunities. Conversely, regions including most of Anhui, northern Jiangsu, and southwestern Zhejiang face structural disadvantages in talent competition due to constraints on local fiscal capacity and geographical conditions.

### 3.5 Health resource agglomeration degree-based equity analysis

From the HRAD perspective, Anhui Province has an HRAD of less than 1, indicating that the fairness of health resource allocation among its geographical regions is relatively poor. In Zhejiang Province, the HRAD for healthcare professionals and registered nurses is less than 1, while the HRAD for licensed (assistant) physicians is greater than 1, suggesting that the fairness of health human resource allocation related to healthcare professionals and registered nurses across Zhejiang Province’s geographical regions needs improvement. Jiangsu Province and Shanghai Municipality have HRAD values above 1, indicating that the distribution of health resources among different geographical regions in these areas is more equitable. The equity in the distribution of health resources among different geographical regions in Shanghai Municipality is superior to that in other regions.

According to the distribution of the population, HRAD/PAD shows that Shanghai and Zhejiang have a relatively good distribution of health human resources. The proportion of health technicians in Jiangsu Province is greater than 1, while the proportions of licensed (assistant) physicians and registered nurses are between 0.9 and 1, indicating that Jiangsu Province needs to improve its fairness. The HRAD/PAD in Anhui Province is less than 1, indicating that Anhui Province still has considerable room for improvement in terms of fairness. This may be due to the contradiction between the development disparities among prefecture-level cities within Jiangsu Province and the allocation of health resources (see Tables 4–6 and Supplementary Tables 10–18).

**Table 4:** The health resource agglomeration degree of health technicians staffing by population in Zhejiang, 2014–2022.

Year	HRAD	PAD	HRAD/PAD
2014	0.923	0.781	1.182
2015	0.943	0.78	1.209
2016	0.944	0.777	1.214
2017	0.949	0.78	1.216
2018	0.933	0.777	1.20
2019	0.941	0.779	1.207
2020	0.923	0.775	1.191
2021	0.933	0.779	1.185
2022	0.943	0.780	1.209

*Prefecture-level city GDP (billion yuan), permanent population (10,000 people), land area (km^2^), and health personnel classification data are sourced from the health statistics data of the Zhejiang Health Commission and the 2015–2023 Statistical Yearbooks of Yangtze River Delta.

**Table 5:** The health resource agglomeration degree of licensed (assistant) physicians by population in Zhejiang, 2014–2022.

Year	HRAD	PAD	HRAD/PAD
2014	1.022	0.781	1.308
2015	1.041	0.78	1.334
2016	1.034	0.777	1.330
2017	1.044	0.78	1.338
2018	1.033	0.777	1.329
2019	1.038	0.779	1.332
2020	1.007	0.775	1.299
2021	1.033	0.779	1.293
2022	1.049	0.780	1.344

*Prefecture-level city GDP (billion yuan), permanent population (10,000 people), land area (km^2^), and health personnel classification data are sourced from the health statistics data of the Zhejiang Health Commission and the 2015–2023 Statistical Yearbooks of Yangtze River Delta.

**Table 6:** The health resource agglomeration degree of registered nurses by population in Zhejiang, 2014–2022.

Year	HRAD	PAD	HRAD/PAD
2014	0.964	0.781	1.234
2015	0.984	0.78	1.261
2016	0.986	0.777	1.268
2017	0.991	0.780	1.270
2018	0.968	0.779	1.245
2019	0.987	0.779	1.267
2020	0.964	0.775	1.243
2021	0.978	0.779	1.238
2022	0.988	0.780	1.265

*Prefecture-level city GDP (billion yuan), permanent population (10,000 people), land area (km^2^), and health personnel classification data are sourced from the health statistics data of the Zhejiang Health Commission and the 2015–2023 Statistical Yearbooks of Yangtze River Delta.

We have constructed a comprehensive comparison table across regions and resource types (see Supplementary Tables 19–22).

### 3.6 Fairness trend analysis

Trend analysis indicates that Zhejiang Province has seen slight improvements in the geographical distribution of licensed physicians. Jiangsu Province has experienced significant changes in geographical distribution (e.g., the Theil index for medical personnel *β* = 0.0034, *p* < 0.001), and there have been fluctuations in GDP distribution equity, necessitating attention to the structure of resource allocation. Shanghai has maintained relatively stable trends across various indicators. Anhui Province has seen a significant improvement in fairness in population distribution (e.g., the Gini coefficient for registered nurses, *β* = −0.0124, *p* < 0.001), but its geographical fairness still faces challenges. Detailed data can be found in Supplementary Tables 23–28.

## 4 Discussion

Research indicates that the allocation of healthcare human resources in the Yangtze River Delta region exhibits the typical characteristics of “overall growth, structural imbalance, geographical disparity, and intra-regional variation as the dominant factor.” Specifically, the total amount of health human resources in the Yangtze River Delta region has been improving, and the number of health technicians, licensed (assistant) physicians, registered nurses and the healthcare ratio per 10,000 people have been increasing, indicating that the growing demand for multi-level and diversified healthcare services from the people has been gradually met. However, the Opinions on Promoting the High-Quality Development of Public Hospitals clearly stated that the healthcare ratio should reach 1:2 (19, 20), and the current healthcare ratio in the Yangtze River Delta region still has a certain gap according to this, indicating that the internal structure of health resources needs to be further adjusted. The reason for this situation may be that with the ageing of China’s population (21, 22).

In view of the above reasons, the Yangtze River Delta region must adopt a dual-pronged strategy: firstly, leveraging its educational strengths (23), it should expand the scale of talent cultivation and enhance professional standards to optimise the overall calibre of its workforce; secondly, it must reform remuneration systems and broaden career progression pathways, thereby establishing a robust foundation for the coordinated and optimised development of healthcare professionals.

Meanwhile, geographic inequity has been found in previous studies to remain an unavoidable problem in the allocation of health human resources (24). This problem exists not only in China but also in other developing countries. For example, in India (25) and Mexico (26), inequality also exists in the geographical distribution of health workers. The World Health Organization’s report also notes that the healthcare industry is facing severe human resource challenges due to the impact of the COVID-19 pandemic. According to a 2023 report, at least 55 countries/regions are currently experiencing severe shortages of healthcare personnel. This issue is particularly severe in Africa, where 37 countries are struggling to address this shortage, which threatens their ability to achieve universal health coverage by 2030 (27, 28). This article comprehensively assesses the equity of health human resource allocation in the Yangtze River Delta region from different perspectives by integrating three methods to provide data support and theoretical basis for optimizing health resource allocation. Research findings indicate that when categorised by geographical region, the Gini coefficient and Theil index results for healthcare human resource allocation exceed those derived from population and GDP-based divisions. Moreover, intra-regional variation proves predominant, a conclusion robustly supported by substantial statistical evidence. Additionally, this result is consistent with the research findings of Ma (29), Shao (27), and Zhou (30). This provides greater confirmation of the effectiveness of the current distribution policy, which is primarily based on demographic factors, in achieving fundamental fairness. However, it also highlights that relying alone on population indicators may prove insufficient to overcome inequalities stemming from geographical and economic factors.

The health resource agglomeration degree not only reflects the efficiency of human resource allocation, but also directly affects the accessibility and equity of medical services. Research shows that there is an imbalance in the distribution of health resources in the Yangtze River Delta region. The reasons for this may include: insufficient accessibility of medical services (31) and the slowing effect of external shocks such as the COVID-19 pandemic on balanced development (32). An example is Anhui Province, where the healthcare human resource concentration (HRAD) has remained at less than 1, reflecting that equity still needs to be improved. This situation is related to the province’s relatively underdeveloped economic status within the Yangtze River Delta region. At the same time, the complex terrain of southern Anhui Province limits the development of transportation networks, further restricting the accessibility of resources. A deeper reason lies in the differences in provincial financial investment between regions, and the lack of attractiveness of grassroots healthcare positions, which exacerbates the outflow of healthcare workers. In contrast, Zhejiang Province prioritises universal healthcare coverage in its allocation of healthcare human resources but has relatively insufficient consideration of geographical factors, This focus may weaken service accessibility in remote areas and impact local capacity to respond to public health emergencies. Additionally, the outbreak of the COVID-19 pandemic has further exacerbated challenges in resource allocation. The pandemic may have reinforced the resource concentration effect in core cities such as Shanghai, Hangzhou, and Nanjing, while also accelerating the outflow of healthcare talent from economically underdeveloped regions, making regional imbalances more complex.

Therefore, it is recommended that the government take into account factors such as population, GDP, and geographical region when allocating resources in order to improve the accessibility of medical personnel services. Specifically, the most pressing priority at present is addressing geographical equity, with particular emphasis on alleviating inadequate access to healthcare resources caused by complex terrain and poor transport links (33). To mitigate this contradiction of ‘the worst geographical equity’, the requirements outlined in the ‘Opinions on Further Improving the Medical and Health Service System’ and other policy documents should be implemented, driving the establishment of urban medical consortiums and county-level healthcare communities. Optimise the spatial distribution of healthcare and health service institutions at all levels and categories. Implement policies to ‘sink’ high-quality medical resources and facilitate the free movement of medical personnel (34). Establish a support mechanism primarily based on ‘one-to-one’ partnerships supplemented by ‘one-to-many’ arrangements. Prioritise the deployment of highly qualified, specialised management and medical personnel to provide administrative and technical support to lower-tier institutions. This will drive the sinking of quality medical resources and enable rational spatial planning of medical and human resources (35). For example, in view of geographical imbalances, Shanghai has adopted the ‘15-min healthcare access zone’ approach to facilitate the sinking of medical resources, based on its own characteristics. Zhejiang Province’s ‘Mountain-Sea’ Healthcare Enhancement Project, by strengthening collaborative support between the province’s developed regions and mountainous and island counties, has also alleviated to some extent the resource allocation imbalance. Secondly, To address the intra-regional variation identified in the research, leveraging the establishment of the National Regional Medical Centres, a regional talent linkage network centred on core cities such as Shanghai, Hangzhou, and Nanjing shall be established. This will enhance mechanisms for two-way talent mobility and multi-site practice across regions (36), thereby facilitating the orderly flow and balanced distribution of high-quality medical human resources within provincial boundaries. Furthermore, the government should progressively leverage modern technologies such as artificial intelligence to empower telemedicine services. Establishing online medical diagnosis platforms would mitigate the impact of economic and geographical factors, optimise resource allocation, and enhance the coverage and efficiency of healthcare services (37).

Although this paper analyses health human resources, it has some limitations. First, the paper mainly focuses on local Chinese data and does not sufficiently discuss international cutting-edge equity assessment frameworks. Second, the paper does not discuss in detail the specific circumstances of establishment, redundancy, contract systems, and labour dispatch. This limits the formulation of policy recommendations on job allocation in health human resource allocation to a certain extent. Third, the classification of the research population is relatively vague, failing to distinguish between employed and unemployed personnel, which may obscure the internal structural inequality of health resources. Fourth, although using the permanent population as the denominator is consistent with planning practices, it does not quantify the impact of cross-city medical treatment. As a highly integrated region, the Yangtze River Delta sees widespread cross-city medical treatment among patients. This means that the HRAD value of a city may overestimate the accessibility of its resources to local residents or underestimate the actual service burden of its resources. In addition, the calculation of HRAD is susceptible to confounding factors such as economic indicators and transportation accessibility. Finally, the fairness of health human resources in the Yangtze River Delta region is related to the level of economic development in each region, but this point is not further researched in this article. As a next step, we can consider collecting data on health human resources and staffing, and fully consider the impact of factors such as population and economy on fairness, in order to more accurately propose countermeasures to promote the distribution of health resources in the Yangtze River Delta region.

## 5 Conclusion

The study shows that the allocation of healthcare human resources in the Yangtze River Delta region is influenced by three overlapping factors: population density, economic development gradients, and geographical accessibility. This has led to a concentration of resources, highlighting shortcomings in regional equity. In response to ‘Healthy China 2030’, the Yangtze River Delta region should establish pilot programmes for the mutual exchange of healthcare professionals among provinces, supplementing policies to accommodate local characteristics, promoting the mobility of local healthcare professionals, overcoming geographical and economic barriers, and promoting the balanced development of resources within the region. The experiences gained from the Yangtze River Delta should be transformed into a governance model that can be replicated nationwide.

## Data Availability

The original contributions presented in the study are included in the article/Supplementary material, further inquiries can be directed to the corresponding author.
