# Supplementary material for: Research on the equity of health manpower resource allocation in the Yangtze River Delta region
Source: Front Public Health. 2025 Oct 14;13:1650147. doi: 10.3389/fpubh.2025.1650147 (PMC12558889; doi:10.3389/fpubh.2025.1650147)
Supplement: Supplementary file 1 [file Data_Sheet_1.docx]

| **Supplementary table 1 The Gini coefficients for the three categories of human resources for health by population in Jiangsu, 2014-2022.** | | | | | |
| --- | --- | --- | --- | --- | --- |
| **Province** | **Year** | | **Health technicians** | **licensed (assistant) physicians** | **Registered nurses** |
| Jiangsu | Early | 2014 | 0.053 | 0.060 | 0.093 |
|  |  | 2015 | 0.057 | 0.051 | 0.089 |
|  |  | 2016 | 0.066 | 0.045 | 0.095 |
|  | Mid | 2017 | 0.071 | 0.055 | 0.098 |
|  |  | 2018 | 0.083 | 0.066 | 0.105 |
|  |  | 2019 | 0.091 | 0.072 | 0.110 |
|  | Late | 2020 | 0.068 | 0.055 | 0.084 |
|  |  | 2021 | 0.071 | 0.056 | 0.082 |
|  |  | 2022 | 0.071 | 0.055 | 0.077 |
| *Prefecture-level city GDP (billion yuan), permanent population (ten thousand people), land area (km²), and health personnel classification data are sourced from the health statistics data of the Zhejiang Health Commission and the 2015-2023 Statistical Yearbooks of Yangtze River Delta. | | | | | |
|  |  |  |  |  |  |
| **Supplementary table 2 The Gini coefficients for the three categories of human resources for health by population in Shanghai, 2014-2022.** | | | | | |
| **Province** | **Year** | | **Health technicians** | **licensed (assistant) physicians** | **Registered nurses** |
| Shanghai | Early | 2014 | 0.332 | 0.311 | 0.349 |
|  |  | 2015 | 0.335 | 0.217 | 0.354 |
|  |  | 2016 | 0.323 | 0.305 | 0.344 |
|  | Mid | 2017 | 0.321 | 0.304 | 0.344 |
|  |  | 2018 | 0.351 | 0.333 | 0.378 |
|  |  | 2019 | 0.343 | 0.326 | 0.367 |
|  | Late | 2020 | 0.347 | 0.326 | 0.371 |
|  |  | 2021 | 0.360 | 0.343 | 0.383 |
|  |  | 2022 | 0.371 | 0.353 | 0.394 |
| *Prefecture-level city GDP (billion yuan), permanent population (ten thousand people), land area (km²), and health personnel classification data are sourced from the health statistics data of the Zhejiang Health Commission and the 2015-2023 Statistical Yearbooks of Yangtze River Delta. | | | | | |
|  |  |  |  |  |  |
| **Supplementary table 3 The Gini coefficients for the three categories of human resources for health by population in Anhui, 2014-2022.** | | | | | |
| **Province** | **Year** | | **Health technicians** | **licensed (assistant) physicians** | **Registered nurses** |
| Anhui | Early | 2014 | 0.133 | 0.122 | 0.181 |
|  |  | 2015 | 0.115 | 0.114 | 0.150 |
|  |  | 2016 | 0.114 | 0.107 | 0.145 |
|  | Mid | 2017 | 0.118 | 0.108 | 0.150 |
|  |  | 2018 | 0.113 | 0.104 | 0.141 |
|  |  | 2019 | 0.120 | 0.111 | 0.149 |
|  | Late | 2020 | 0.071 | 0.058 | 0.093 |
|  |  | 2021 | 0.067 | 0.055 | 0.084 |
|  |  | 2022 | 0.053 | 0.052 | 0.070 |
| *Prefecture-level city GDP (billion yuan), permanent population (ten thousand people), land area (km²), and health personnel classification data are sourced from the health statistics data of the Zhejiang Health Commission and the 2015-2023 Statistical Yearbooks of Yangtze River Delta. | | | | | |

| **Supplementary table 4 The Gini coefficients for the three categories of human resources for health by geographic area in Jiangsu, 2014-2022.** | | | | | |
| --- | --- | --- | --- | --- | --- |
| **Province** | **Year** | | **Health technicians** | **licensed (assistant) physicians** | **Registered nurses** |
| Jiangsu | Early | 2014 | 0.244 | 0.248 | 0.277 |
|  |  | 2015 | 0.248 | 0.240 | 0.274 |
|  |  | 2016 | 0.259 | 0.243 | 0.282 |
|  | Mid | 2017 | 0.266 | 0.254 | 0.288 |
|  |  | 2018 | 0.280 | 0.266 | 0.297 |
|  |  | 2019 | 0.288 | 0.270 | 0.300 |
|  | Late | 2020 | 0.290 | 0.273 | 0.296 |
|  |  | 2021 | 0.302 | 0.285 | 0.304 |
|  |  | 2022 | 0.305 | 0.289 | 0.305 |
| *Prefecture-level city GDP (billion yuan), permanent population (ten thousand people), land area (km²), and health personnel classification data are sourced from the health statistics data of the Zhejiang Health Commission and the 2015-2023 Statistical Yearbooks of Yangtze River Delta. | | | | | |
| **Supplementary table 5 The Gini coefficients for the three categories of human resources for health by geographic area in Shanghai, 2014-2022.** | | | | | |
| **Province** | **Year** | | **Health technicians** | **licensed (assistant) physicians** | **Registered nurses** |
| Shanghai | Early | 2014 | 0.660 | 0.641 | 0.677 |
|  |  | 2015 | 0.659 | 0.642 | 0.677 |
|  |  | 2016 | 0.668 | 0.650 | 0.687 |
|  | Mid | 2017 | 0.667 | 0.653 | 0.689 |
|  |  | 2018 | 0.686 | 0.668 | 0.710 |
|  |  | 2019 | 0.680 | 0.663 | 0.703 |
|  | Late | 2020 | 0.674 | 0.655 | 0.696 |
|  |  | 2021 | 0.674 | 0.657 | 0.695 |
|  |  | 2022 | 0.672 | 0.656 | 0.691 |
| *Prefecture-level city GDP (billion yuan), permanent population (ten thousand people), land area (km²), and health personnel classification data are sourced from the health statistics data of the Zhejiang Health Commission and the 2015-2023 Statistical Yearbooks of Yangtze River Delta. | | | | | |
|  |  |  |  |  |  |
| **Supplementary table 6 The Gini coefficients for the three categories of human resources for health by geographic area in Anhui, 2014-2022.** | | | | | |
| **Province** | **Year** | | **Health technicians** | **licensed (assistant) physicians** | **Registered nurses** |
| Anhui | Early | 2014 | 0.281 | 0.269 | 0.309 |
|  |  | 2015 | 0.283 | 0.268 | 0.306 |
|  |  | 2016 | 0.303 | 0.288 | 0.328 |
|  | Mid | 2017 | 0.305 | 0.294 | 0.329 |
|  |  | 2018 | 0.311 | 0.301 | 0.332 |
|  |  | 2019 | 0.319 | 0.309 | 0.337 |
|  | Late | 2020 | 0.309 | 0.300 | 0.323 |
|  |  | 2021 | 0.310 | 0.303 | 0.322 |
|  |  | 2022 | 0.309 | 0.302 | 0.322 |
| *Prefecture-level city GDP (billion yuan), permanent population (ten thousand people), land area (km²), and health personnel classification data are sourced from the health statistics data of the Zhejiang Health Commission and the 2015-2023 Statistical Yearbooks of Yangtze River Delta. | | | | | |

| **Supplementary table 7 The Gini coefficients for the three categories of human resources for health by GDP* in Jiangsu, 2014-2022.** | | | | | |
| --- | --- | --- | --- | --- | --- |
| **Province** | **Year** | | **Health technicians** | **licensed (assistant) physicians** | **Registered nurses** |
| Jiangsu | Early | 2014 | 0.197 | 0.170 | 0.178 |
|  |  | 2015 | 0.184 | 0.171 | 0.167 |
|  |  | 2016 | 0.176 | 0.172 | 0.167 |
|  | Mid | 2017 | 0.167 | 0.164 | 0.156 |
|  |  | 2018 | 0.170 | 0.167 | 0.168 |
|  |  | 2019 | 0.158 | 0.164 | 0.163 |
|  | Late | 2020 | 0.155 | 0.161 | 0.165 |
|  |  | 2021 | 0.149 | 0.153 | 0.160 |
|  |  | 2022 | 0.145 | 0.149 | 0.156 |
| * GDP: Gross Domestic Product  *Prefecture-level city GDP (billion yuan), permanent population (ten thousand people), land area (km²), and health personnel classification data are sourced from the health statistics data of the Zhejiang Health Commission and the 2015-2023 Statistical Yearbooks of Yangtze River Delta. | | | | | |
| **Supplementary table 8 The Gini coefficients for the three categories of human resources for health by GDP* in Shanghai, 2014-2022.** | | | | | |
| **Province** | **Year** | | **Health technicians** | **licensed (assistant) physicians** | **Registered nurses** |
| Shanghai | Early | 2014 | 0.306 | 0.287 | 0.320 |
|  |  | 2015 | 0.310 | 0.295 | 0.325 |
|  |  | 2016 | 0.311 | 0.295 | 0.325 |
|  | Mid | 2017 | 0.326 | 0.310 | 0.342 |
|  |  | 2018 | 0.346 | 0.330 | 0.363 |
|  |  | 2019 | 0.319 | 0.301 | 0.337 |
|  | Late | 2020 | 0.307 | 0.285 | 0.324 |
|  |  | 2021 | 0.324 | 0.303 | 0.342 |
|  |  | 2022 | 0.322 | 0.303 | 0.339 |
| * GDP: Gross Domestic Product  *Prefecture-level city GDP (billion yuan), permanent population (ten thousand people), land area (km²), and health personnel classification data are sourced from the health statistics data of the Zhejiang Health Commission and the 2015-2023 Statistical Yearbooks of Yangtze River Delta. | | | | | |

| **Supplementary table 9 The Gini coefficients for the three categories of human resources for health by GDP* in Anhui, 2014-2022.** | | | | | |
| --- | --- | --- | --- | --- | --- |
| **Province** | **Year** | | **Health technicians** | **licensed (assistant) physicians** | **Registered nurses** |
| Anhui | Early | 2014 | 0.187 | 0.198 | 0.156 |
|  |  | 2015 | 0.205 | 0.213 | 0.172 |
|  |  | 2016 | 0.200 | 0.208 | 0.183 |
|  | Mid | 2017 | 0.198 | 0.206 | 0.180 |
|  |  | 2018 | 0.206 | 0.209 | 0.193 |
|  |  | 2019 | 0.163 | 0.166 | 0.146 |
|  | Late | 2020 | 0.188 | 0.195 | 0.179 |
|  |  | 2021 | 0.195 | 0.201 | 0.187 |
|  |  | 2022 | 0.204 | 0.207 | 0.195 |
| * GDP: Gross Domestic Product  *Prefecture-level city GDP (billion yuan), permanent population (ten thousand people), land area (km²), and health personnel classification data are sourced from the health statistics data of the Zhejiang Health Commission and the 2015-2023 Statistical Yearbooks of Yangtze River Delta. | | | | | |
