# Supplementary material for: Research on the equity of health manpower resource allocation in the Yangtze River Delta region
Source: Front Public Health. 2025 Oct 14;13:1650147. doi: 10.3389/fpubh.2025.1650147 (PMC12558889; doi:10.3389/fpubh.2025.1650147)
Supplement: Supplementary file 2 [file Data_Sheet_2.docx]

| Supplementary table 10 The Health Resource Agglomeration Degree of health technician staffing by population in Jiangsu, 2014-2022. | | | | |
| --- | --- | --- | --- | --- |
| **Year** | | **HRAD** | **PAD** | **HRAD/PAD** |
| **Early** | **2014** | 1.428 | 1.263 | 1.131 |
|  | **2015** | 1.421 | 1.260 | 1.128 |
|  | **2016** | 1.413 | 1.253 | 1.128 |
| **Mid** | **2017** | 1.412 | 1.250 | 1.129 |
|  | **2018** | 1.416 | 1.249 | 1.134 |
|  | **2019** | 1.411 | 1.246 | 1.133 |
| **Late** | **2020** | 1.385 | 1.295 | 1.070 |
|  | **2021** | 1.362 | 1.289 | 1.075 |
|  | **2022** | 1.335 | 1.289 | 1.036 |
| *Prefecture-level city GDP (billion yuan), permanent population (ten thousand people), land area (km²), and health personnel classification data are sourced from the health statistics data of the Zhejiang Health Commission and the 2015-2023 Statistical Yearbooks of Yangtze River Delta. | | | | |
|  |  |  |  |  |
| **Supplementary table 11 The Health Resource Agglomeration Degree of licensed (assistant) physicians by population in Jiangsu, 2014-2022.** | | | | |
| **Year** | | **HRAD** | **PAD** | **HRAD/PAD** |
| **Early** | **2014** | 1.237 | 1.263 | 0.979 |
|  | **2015** | 1.237 | 1.260 | 0.982 |
|  | **2016** | 1.258 | 1.253 | 1.004 |
| **Mid** | **2017** | 1.251 | 1.250 | 1.000 |
|  | **2018** | 1.261 | 1.249 | 1.010 |
|  | **2019** | 1.269 | 1.246 | 1.019 |
| **Late** | **2020** | 1.237 | 1.295 | 0.955 |
|  | **2021** | 1.199 | 1.289 | 0.960 |
|  | **2022** | 1.177 | 1.289 | 0.913 |
| *Prefecture-level city GDP (billion yuan), permanent population (ten thousand people), land area (km²), and health personnel classification data are sourced from the health statistics data of the Zhejiang Health Commission and the 2015-2023 Statistical Yearbooks of Yangtze River Delta. | | | | |
|  |  |  |  |  |
|  |  |  |  |  |
| **Supplementary table 12 The Health Resource Agglomeration Degree of registered nurse by population in Jiangsu, 2014-2022.** | | | | |
| **Year** | | **HRAD** | **PAD** | **HRAD/PAD** |
| **Early** | **2014** | 1.237 | 1.263 | 0.980 |
|  | **2015** | 1.238 | 1.260 | 0.982 |
|  | **2016** | 1.236 | 1.253 | 0.986 |
| **Mid** | **2017** | 1.235 | 1.250 | 0.988 |
|  | **2018** | 1.249 | 1.249 | 1.000 |
|  | **2019** | 1.241 | 1.246 | 0.996 |
| **Late** | **2020** | 1.215 | 1.295 | 0.939 |
|  | **2021** | 1.196 | 1.289 | 0.943 |
|  | **2022** | 1.167 | 1.289 | 0.905 |
| *Prefecture-level city GDP (billion yuan), permanent population (ten thousand people), land area (km²), and health personnel classification data are sourced from the health statistics data of the Zhejiang Health Commission and the 2015-2023 Statistical Yearbooks of Yangtze River Delta. | | | | |

| **Supplementary table 13 The Health Resource Agglomeration Degree of health technician staffing by population in Shanghai, 2014-2022.** | | | | |
| --- | --- | --- | --- | --- |
| **Year** | | **HRAD** | **PAD** | **HRAD/PAD** |
| **Early** | **2014** | 6.375 | 6.228 | 1.024 |
|  | **2015** | 6.258 | 6.18 | 1.013 |
|  | **2016** | 6.426 | 6.332 | 1.015 |
| **Mid** | **2017** | 6.397 | 6.288 | 1.017 |
|  | **2018** | 6.602 | 6.278 | 1.052 |
|  | **2019** | 6.393 | 6.259 | 1.021 |
| **Late** | **2020** | 6.361 | 6.346 | 1.000 |
|  | **2021** | 6.432 | 6.344 | 1.000 |
|  | **2022** | 6.318 | 6.032 | 1.047 |
| *Prefecture-level city GDP (billion yuan), permanent population (ten thousand people), land area (km²), and health personnel classification data are sourced from the health statistics data of the Zhejiang Health Commission and the 2015-2023 Statistical Yearbooks of Yangtze River Delta. | | | | |
|  |  |  |  |  |
| **Supplementary table 14 The Health Resource Agglomeration Degree of licensed (assistant) physicians by population in Shanghai, 2014-2022.** | | | | |
| **Year** | | **HRAD** | **PAD** | **HRAD/PAD** |
| **Early** | **2014** | 6.823 | 6.228 | 1.096 |
|  | **2015** | 6.684 | 6.18 | 1.081 |
|  | **2016** | 6.66 | 6.332 | 1.052 |
| **Mid** | **2017** | 6.652 | 6.288 | 1.044 |
|  | **2018** | 6.759 | 6.278 | 1.077 |
|  | **2019** | 6.464 | 6.259 | 1.033 |
| **Late** | **2020** | 6.349 | 6.346 | 1.000 |
|  | **2021** | 6.436 | 6.344 | 1.001 |
|  | **2022** | 6.303 | 6.302 | 1.000 |
| *Prefecture-level city GDP (billion yuan), permanent population (ten thousand people), land area (km²), and health personnel classification data are sourced from the health statistics data of the Zhejiang Health Commission and the 2015-2023 Statistical Yearbooks of Yangtze River Delta. | | | | |
|  |  |  |  |  |
|  |  |  |  |  |
| **Supplementary table 15 The Health Resource Agglomeration Degree of registered nurse by population in Shanghai, 2014-2022.** | | | | |
| **Year** | | **HRAD** | **PAD** | **HRAD/PAD** |
| **Early** | **2014** | 7.551 | 6.228 | 1.212 |
|  | **2015** | 7.326 | 6.18 | 1.185 |
|  | **2016** | 7.41 | 6.332 | 1.17 |
| **Mid** | **2017** | 7.311 | 6.288 | 1.163 |
|  | **2018** | 7.497 | 6.278 | 1.194 |
|  | **2019** | 7.19 | 6.259 | 1.149 |
| **Late** | **2020** | 7.109 | 6.346 | 1.12 |
|  | **2021** | 7.079 | 6.344 | 1.121 |
|  | **2022** | 6.871 | 6.302 | 1.09 |
| *Prefecture-level city GDP (billion yuan), permanent population (ten thousand people), land area (km²), and health personnel classification data are sourced from the health statistics data of the Zhejiang Health Commission and the 2015-2023 Statistical Yearbooks of Yangtze River Delta. | | | | |

| **Supplementary table 16 The Health Resource Agglomeration Degree of health technician staffing by population in Anhui, 2014-2022.** | | | | |
| --- | --- | --- | --- | --- |
| **Year** | | **HRAD** | **PAD** | **HRAD/PAD** |
| **Early** | **2014** | 0.491 | 0.729 | 0.674 |
|  | **2015** | 0.487 | 0.734 | 0.664 |
|  | **2016** | 0.484 | 0.734 | 0.66 |
| **Mid** | **2017** | 0.483 | 0.736 | 0.656 |
|  | **2018** | 0.483 | 0.741 | 0.651 |
|  | **2019** | 0.490 | 0.743 | 0.66 |
| **Late** | **2020** | 0.524 | 0.705 | 0.744 |
|  | **2021** | 0.529 | 0.71 | 0.744 |
|  | **2022** | 0.547 | 0.706 | 0.775 |
| *Prefecture-level city GDP (billion yuan), permanent population (ten thousand people), land area (km²), and health personnel classification data are sourced from the health statistics data of the Zhejiang Health Commission and the 2015-2023 Statistical Yearbooks of Yangtze River Delta. | | | | |
|  |  |  |  |  |
| **Supplementary table 17 The Health Resource Agglomeration Degree of licensed (assistant) physicians by population in Anhui, 2014-2022.** | | | | |
| **Year** | | **HRAD** | **PAD** | **HRAD/PAD** |
| **Early** | **2014** | 0.542 | 0.729 | 0.744 |
|  | **2015** | 0.534 | 0.734 | 0.728 |
|  | **2016** | 0.523 | 0.734 | 0.713 |
| **Mid** | **2017** | 0.526 | 0.736 | 0.714 |
|  | **2018** | 0.517 | 0.741 | 0.698 |
|  | **2019** | 0.521 | 0.734 | 0.701 |
| **Late** | **2020** | 0.574 | 0.705 | 0.814 |
|  | **2021** | 0.578 | 0.705 | 0.814 |
|  | **2022** | 0.589 | 0.706 | 0.834 |
| *Prefecture-level city GDP (billion yuan), permanent population (ten thousand people), land area (km²), and health personnel classification data are sourced from the health statistics data of the Zhejiang Health Commission and the 2015-2023 Statistical Yearbooks of Yangtze River Delta. | | | | |
|  |  |  |  |  |
|  |  |  |  |  |
| **Supplementary table 18 The Health Resource Agglomeration Degree of registered nurse by population in Anhui, 2014-2022.** | | | | |
| **Year** | | **HRAD** | **PAD** | **HRAD/PAD** |
| **Early** | **2014** | 0.552 | 0.729 | 0.758 |
|  | **2015** | 0.547 | 0.734 | 0.746 |
|  | **2016** | 0.543 | 0.734 | 0.74 |
| **Mid** | **2017** | 0.544 | 0.736 | 0.739 |
|  | **2018** | 0.542 | 0.741 | 0.731 |
|  | **2019** | 0.547 | 0.743 | 0.737 |
| **Late** | **2020** | 0.588 | 0.705 | 0.834 |
|  | **2021** | 0.592 | 0.705 | 0.834 |
|  | **2022** | 0.617 | 0.706 | 0.874 |
| *Prefecture-level city GDP (billion yuan), permanent population (ten thousand people), land area (km²), and health personnel classification data are sourced from the health statistics data of the Zhejiang Health Commission and the 2015-2023 Statistical Yearbooks of Yangtze River Delta. | | | | |
