# Supplementary material for: Research on the equity of health manpower resource allocation in the Yangtze River Delta region
Source: Front Public Health. 2025 Oct 14;13:1650147. doi: 10.3389/fpubh.2025.1650147 (PMC12558889; doi:10.3389/fpubh.2025.1650147)
Supplement: Supplementary file 3 [file Data_Sheet_3.docx]

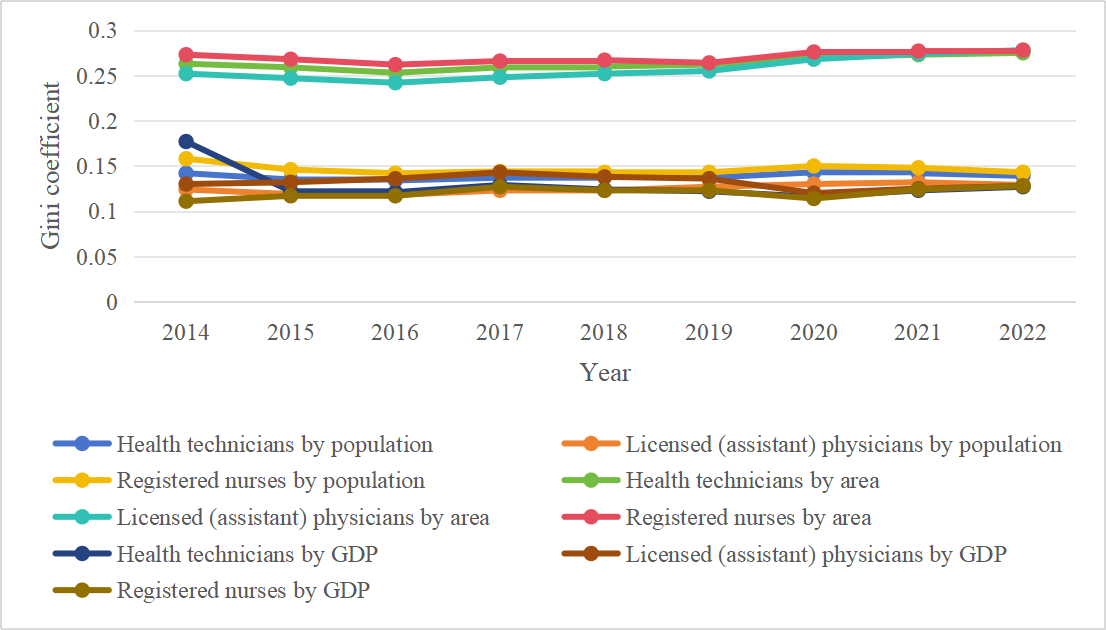


**Supplementary Figure 2** **The Gini coefficients for the three categories of human resources for health in Zhejiang, 2014-2022.**

*Prefecture-level city GDP (billion yuan), permanent population (ten thousand people), land area (km²), and health personnel classification data are sourced from the health statistics data of the Zhejiang Health Commission and the 2015-2023 Statistical Yearbooks of Yangtze River Delta.

**
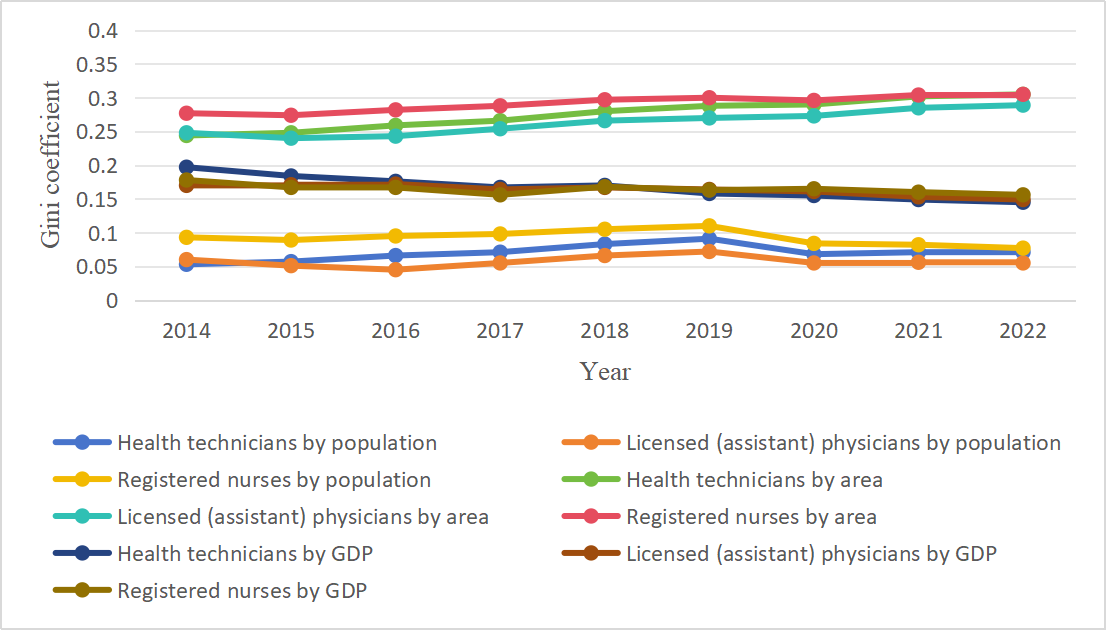
**

**Supplementary Figure 3 The Gini coefficients for the three categories of human resources for health in Jiangsu, 2014-2022.**

*Prefecture-level city GDP (billion yuan), permanent population (ten thousand people), land area (km²), and health personnel classification data are sourced from the health statistics data of the Zhejiang Health Commission and the 2015-2023 Statistical Yearbooks of Yangtze River Delta.

**
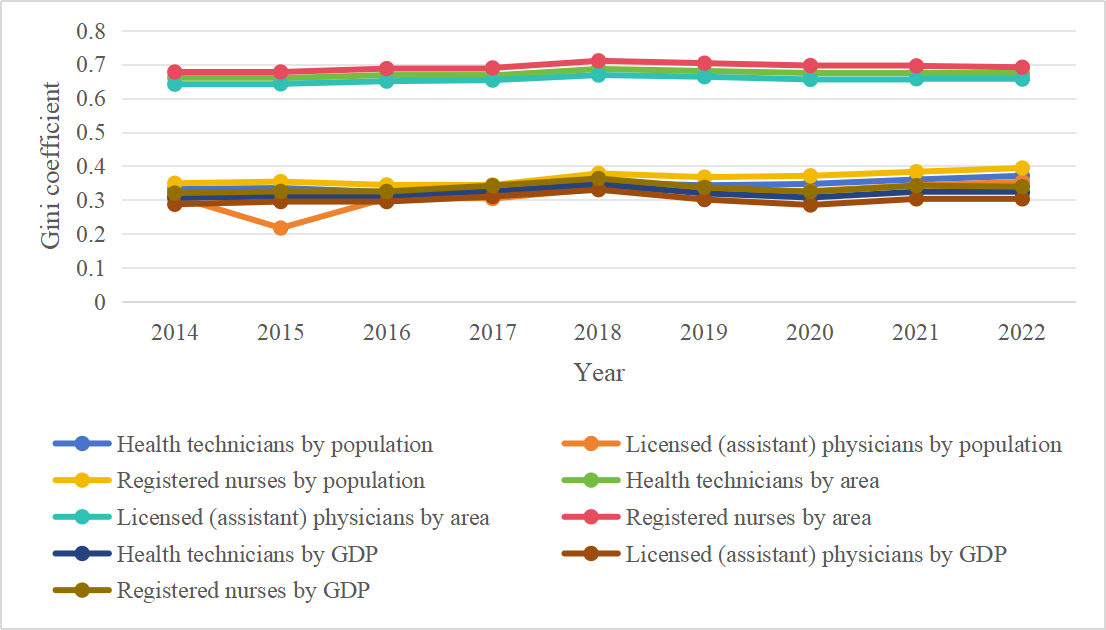
**

**Supplementary Figure 4 The Gini coefficients for the three categories of human resources for health in Shanghai, 2014-2022.**

*Prefecture-level city GDP (billion yuan), permanent population (ten thousand people), land area (km²), and health personnel classification data are sourced from the health statistics data of the Zhejiang Health Commission and the 2015-2023 Statistical Yearbooks of Yangtze River Delta.

**
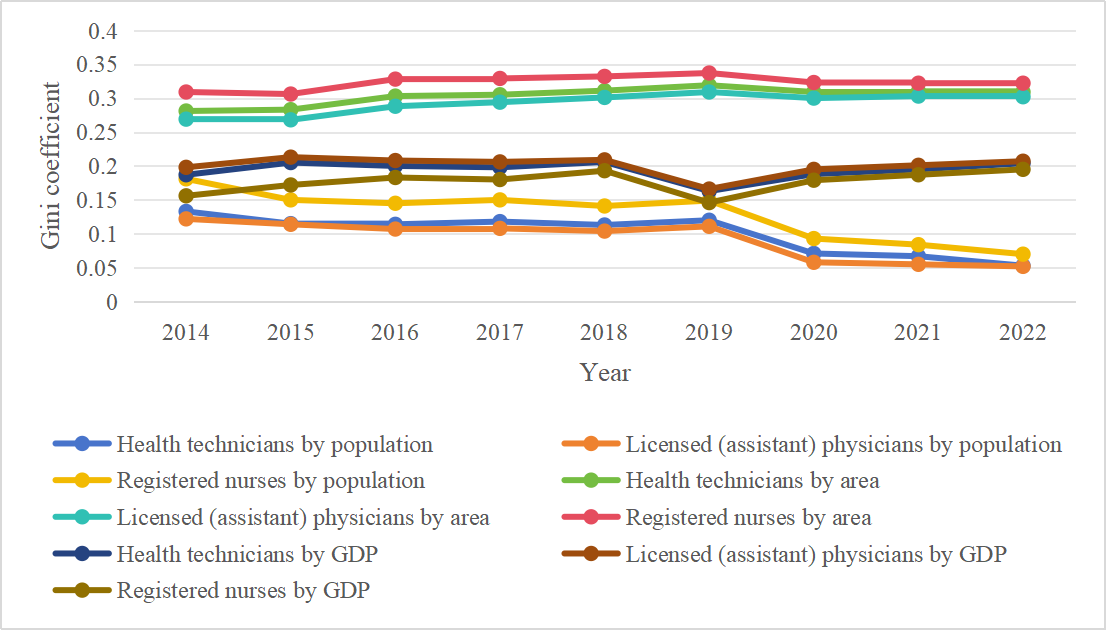
**

**Supplementary Figure 5 The Gini coefficients for the three categories of human resources for health in Anhui, 2014-2022.**

*Prefecture-level city GDP (billion yuan), permanent population (ten thousand people), land area (km²), and health personnel classification data are sourced from the health statistics data of the Zhejiang Health Commission and the 2015-2023 Statistical Yearbooks of Yangtze River Delta.
