# Supplementary material for: Research on the equity of health manpower resource allocation in the Yangtze River Delta region
Source: Front Public Health. 2025 Oct 14;13:1650147. doi: 10.3389/fpubh.2025.1650147 (PMC12558889; doi:10.3389/fpubh.2025.1650147)
Supplement: Supplementary file 4 [file Data_Sheet_4.docx]

We have constructed a comprehensive comparison table across regions and resource types. Although Supplementary Tables 19-22 and Table 3 in the main text measure equity at the provincial and Yangtze River Delta regional levels using the Thiel index, both reveal that equity in geographical distribution is significantly lower than equity in distribution based on population and GDP. This discrepancy stems from the fact that current policy planning primarily considers the impact of population and economic factors on the allocation of healthcare resources, while relatively neglecting the actual inadequacy of resource accessibility in geographically complex regions, leading to insufficient healthcare service accessibility. We should comprehensively consider the three dimensions of population distribution, economy, and geography to establish a multi-dimensional balanced allocation model, thereby optimising resource allocation strategies and narrowing the actual gaps between regions.

In addition, to assess the statistical significance of the above trends, we calculated the *p-values* and 95% confidence intervals for the slopes of each linear regression. The analysis revealed that statistically significant changes (*p < 0.05*) exhibited regular patterns. For example, in terms of fairness in geographical distribution, the Thiel index for healthcare technician and registered nurses in Jiangsu Province showed extremely significant improvements (*β = 0.0034, p < 0.001; β = 0.0017, p < 0.001*). Conversely, most improvements in distribution by population and GDP were also significant, such as the significant decrease in the Gini coefficient for registered nurses in Anhui Province by population distribution (*β = -0.0125, p < 0.001*). These results indicate that the observed trends are not random fluctuations but statistically significant real changes, as shown in Supplementary Tables 23–25. Visualisations of the linear relationships are presented in Supplementary Tables 26–28.

| Supplementary table 19 The Gini coefficient, Theil index, and **Health Resource Agglomeration Degree** of three categories of health human resources in Zhejiang Province from 2014 to 2022. | | | | | | | | | | | |
| --- | --- | --- | --- | --- | --- | --- | --- | --- | --- | --- | --- |
| **health workers** | **Year** | | **Gini coefficients** | | | **Theil Index** | | | **Health Resource Agglomeration Degree** | | |
|  |  |  | **Population-based allocation** | **Geographic area-based allocation** | **GDP-based allocation** | **Population-based allocation** | **Geographic area-based allocation** | **GDP-based allocation** | **HRAD** | **PAD** | **HRAD/PAD** |
| **Health technicians** | Early | 2014 | 0.142 | 0.263 | 0.177 | 0.015 | 0.055 | 0.010 | 1.022 | 0.781 | 1.308 |
|  |  | 2015 | 0.135 | 0.259 | 0.122 | 0.014 | 0.054 | 0.010 | 1.041 | 0.78 | 1.334 |
|  |  | 2016 | 0.134 | 0.253 | 0.121 | 0.014 | 0.051 | 0.010 | 1.034 | 0.777 | 1.330 |
|  | Mid | 2017 | 0.137 | 0.259 | 0.129 | 0.014 | 0.053 | 0.012 | 1.044 | 0.78 | 1.338 |
|  |  | 2018 | 0.138 | 0.260 | 0.124 | 0.014 | 0.054 | 0.011 | 1.033 | 0.777 | 1.329 |
|  |  | 2019 | 0.137 | 0.262 | 0.122 | 0.014 | 0.054 | 0.011 | 1.038 | 0.779 | 1.332 |
|  | Late | 2020 | 0.143 | 0.270 | 0.116 | 0.015 | 0.058 | 0.009 | 1.007 | 0.775 | 1.299 |
|  |  | 2021 | 0.142 | 0.273 | 0.123 | 0.014 | 0.059 | 0.011 | 1.033 | 0.779 | 1.293 |
|  |  | 2022 | 0.139 | 0.275 | 0.127 | 0.013 | 0.059 | 0.011 | 1.049 | 0.780 | 1.344 |
| **licensed (assistant) physicians** | Early | 2014 | 0.124 | 0.252 | 0.130 | 0.012 | 0.052 | 0.012 | 1.022 | 0.781 | 1.308 |
|  |  | 2015 | 0.119 | 0.247 | 0.132 | 0.011 | 0.049 | 0.012 | 1.041 | 0.78 | 1.334 |
|  |  | 2016 | 0.118 | 0.242 | 0.136 | 0.011 | 0.049 | 0.013 | 1.034 | 0.777 | 1.330 |
|  | Mid | 2017 | 0.123 | 0.248 | 0.143 | 0.012 | 0.050 | 0.014 | 1.044 | 0.78 | 1.338 |
|  |  | 2018 | 0.123 | 0.252 | 0.138 | 0.012 | 0.052 | 0.013 | 1.033 | 0.777 | 1.329 |
|  |  | 2019 | 0.127 | 0.255 | 0.136 | 0.012 | 0.053 | 0.013 | 1.038 | 0.779 | 1.332 |
|  | Late | 2020 | 0.130 | 0.268 | 0.120 | 0.012 | 0.057 | 0.010 | 1.007 | 0.775 | 1.299 |
|  |  | 2021 | 0.132 | 0.274 | 0.125 | 0.012 | 0.059 | 0.011 | 1.033 | 0.779 | 1.293 |
|  |  | 2022 | 0.129 | 0.278 | 0.128 | 0.012 | 0.060 | 0.012 | 1.049 | 0.780 | 1.344 |
| **Registered nurses** | Early | 2014 | .0.158 | 0.273 | 0.111 | 0.018 | 0.058 | 0.009 | 0.964 | 0.781 | 1.234 |
|  |  | 2015 | 0.146 | 0.268 | 0.117 | 0.016 | 0.056 | 0.010 | 0.984 | 0.78 | 1.261 |
|  |  | 2016 | 0.142 | 0.262 | 0.117 | 0.016 | 0.054 | 0.010 | 0.986 | 0.777 | 1.268 |
|  | Mid | 2017 | 0.144 | 0.266 | 0.127 | 0.016 | 0.055 | 0.011 | 0.991 | 0.780 | 1.270 |
|  |  | 2018 | 0.143 | 0.267 | 0.123 | 0.016 | 0.056 | 0.011 | 0.968 | 0.779 | 1.245 |
|  |  | 2019 | 0.143 | 0.264 | 0.123 | 0.015 | 0.056 | 0.011 | 0.987 | 0.779 | 1.267 |
|  | Late | 2020 | 0.150 | 0.276 | 0.114 | 0.016 | 0.060 | 0.009 | 0.964 | 0.775 | 1.243 |
|  |  | 2021 | 0.148 | 0.277 | 0.124 | 0.016 | 0.060 | 0.011 | 0.978 | 0.779 | 1.238 |
|  |  | 2022 | 0.143 | 0.278 | 0.128 | 0.014 | 0.061 | 0.012 | 0.988 | 0.780 | 1.265 |
| *Prefecture-level city GDP (billion yuan), permanent population (ten thousand people), land area (km²), and health personnel classification data are sourced from the health statistics data of the Zhejiang Health Commission and the 2015-2023 Statistical Yearbooks of Yangtze River Delta. | | | | | | | | | | | |

| Supplementary table 20 The Gini coefficient, Theil index, and **Health Resource Agglomeration Degree** of three categories of health human resources in Jiangsu Province from 2014 to 2022. | | | | | | | | | | | |
| --- | --- | --- | --- | --- | --- | --- | --- | --- | --- | --- | --- |
| **health workers** | **Year** | | **Gini coefficients** | | | **Theil Index** | | | **Health Resource Agglomeration Degree** | | |
|  |  |  | **Population-based allocation** | **Geographic area-based allocation** | **GDP-based allocation** | **Population-based allocation** | **Geographic area-based allocation** | **GDP-based allocation** | **HRAD** | **PAD** | **HRAD/PAD** |
| **Health technicians** | Early | 2014 | 0.053 | 0.244 | 0.197 | 0.002 | 0.042 | 0.025 | 1.428 | 1.263 | 1.131 |
|  |  | 2015 | 0.057 | 0.248 | 0.184 | 0.002 | 0.044 | 0.030 | 1.421 | 1.260 | 1.128 |
|  |  | 2016 | 0.066 | 0.259 | 0.176 | 0.003 | 0.047 | 0.029 | 1.413 | 1.253 | 1.128 |
|  | Mid | 2017 | 0.071 | 0.266 | 0.167 | 0.004 | 0.051 | 0.028 | 1.412 | 1.250 | 1.129 |
|  |  | 2018 | 0.083 | 0.280 | 0.170 | 0.005 | 0.056 | 0.031 | 1.416 | 1.249 | 1.134 |
|  |  | 2019 | 0.091 | 0.288 | 0.158 | 0.006 | 0.059 | 0.018 | 1.411 | 1.246 | 1.133 |
|  | Late | 2020 | 0.068 | 0.290 | 0.155 | 0.004 | 0.061 | 0.024 | 1.385 | 1.295 | 1.070 |
|  |  | 2021 | 0.071 | 0.302 | 0.149 | 0.004 | 0.066 | 0.026 | 1.362 | 1.289 | 1.075 |
|  |  | 2022 | 0.071 | 0.305 | 0.145 | 0.004 | 0.068 | 0.029 | 1.335 | 1.289 | 1.036 |
| **licensed (assistant) physicians** | Early | 2014 | 0.060 | 0.248 | 0.170 | 0.003 | 0.044 | 0.020 | 1.237 | 1.263 | 0.979 |
|  |  | 2015 | 0.051 | 0.240 | 0.171 | 0.002 | 0.041 | 0.021 | 1.237 | 1.260 | 0.982 |
|  |  | 2016 | 0.045 | 0.243 | 0.172 | 0.002 | 0.042 | 0.021 | 1.258 | 1.253 | 1.004 |
|  | Mid | 2017 | 0.055 | 0.254 | 0.164 | 0.002 | 0.047 | 0.020 | 1.251 | 1.250 | 1.000 |
|  |  | 2018 | 0.066 | 0.266 | 0.167 | 0.003 | 0.051 | 0.021 | 1.261 | 1.249 | 1.010 |
|  |  | 2019 | 0.072 | 0.270 | 0.164 | 0.004 | 0.052 | 0.020 | 1.269 | 1.246 | 1.019 |
|  | Late | 2020 | 0.055 | 0.273 | 0.161 | 0.003 | 0.054 | 0.019 | 1.237 | 1.295 | 0.955 |
|  |  | 2021 | 0.056 | 0.285 | 0.153 | 0.003 | 0.059 | 0.017 | 1.199 | 1.289 | 0.960 |
|  |  | 2022 | 0.055 | 0.289 | 0.149 | 0.003 | 0.061 | 0.016 | 1.177 | 1.289 | 0.913 |
| **Registered nurses** | Early | 2014 | 0.093 | 0.277 | 0.178 | 0.006 | 0.057 | 0.022 | 1.237 | 1.263 | 0.980 |
|  |  | 2015 | 0.089 | 0.274 | 0.167 | 0.006 | 0.055 | 0.021 | 1.238 | 1.260 | 0.982 |
|  |  | 2016 | 0.095 | 0.282 | 0.167 | 0.007 | 0.058 | 0.021 | 1.236 | 1.253 | 0.986 |
|  | Mid | 2017 | 0.098 | 0.288 | 0.156 | 0.007 | 0.061 | 0.019 | 1.235 | 1.250 | 0.988 |
|  |  | 2018 | 0.105 | 0.297 | 0.168 | 0.008 | 0.063 | 0.022 | 1.249 | 1.249 | 1.000 |
|  |  | 2019 | 0.110 | 0.300 | 0.163 | 0.009 | 0.065 | 0.021 | 1.241 | 1.246 | 0.996 |
|  | Late | 2020 | 0.084 | 0.296 | 0.165 | 0.006 | 0.064 | 0.021 | 1.215 | 1.295 | 0.939 |
|  |  | 2021 | 0.082 | 0.304 | 0.160 | 0.005 | 0.068 | 0.020 | 1.196 | 1.289 | 0.943 |
|  |  | 2022 | 0.077 | 0.305 | 0.156 | 0.005 | 0.068 | 0.019 | 1.167 | 1.289 | 0.905 |
| *Prefecture-level city GDP (billion yuan), permanent population (ten thousand people), land area (km²), and health personnel classification data are sourced from the health statistics data of the Zhejiang Health Commission and the 2015-2023 Statistical Yearbooks of Yangtze River Delta. | | | | | | | | | | | |

| Supplementary table 21 The Gini coefficient, Theil index, and **Health Resource Agglomeration Degree** of three categories of health human resources in Shanghai from 2014 to 2022. | | | | | | | | | | | |
| --- | --- | --- | --- | --- | --- | --- | --- | --- | --- | --- | --- |
| **health workers** | **Year** | | **Gini coefficients** | | | **Theil Index** | | | **Health Resource Agglomeration Degree** | | |
|  |  |  | **Population-based allocation** | **Geographic area-based allocation** | **GDP-based allocation** | **Population-based allocation** | **Geographic area-based allocation** | **GDP-based allocation** | **HRAD** | **PAD** | **HRAD/PAD** |
| **Health technicians** | Early | 2014 | 0.332 | 0.660 | 0.306 | 0.106 | 0.537 | 0.066 | 6.375 | 6.228 | 1.024 |
|  |  | 2015 | 0.335 | 0.659 | 0.310 | 0.108 | 0.535 | 0.067 | 6.258 | 6.18 | 1.013 |
|  |  | 2016 | 0.323 | 0.668 | 0.311 | 0.092 | 0.524 | 0.067 | 6.426 | 6.332 | 1.015 |
|  | Mid | 2017 | 0.321 | 0.667 | 0.326 | 0.091 | 0.523 | 0.074 | 6.397 | 6.288 | 1.017 |
|  |  | 2018 | 0.351 | 0.686 | 0.346 | 0.106 | 0.560 | 0.085 | 6.602 | 6.278 | 1.052 |
|  |  | 2019 | 0.343 | 0.680 | 0.319 | 0.099 | 0.545 | 0.071 | 6.393 | 6.259 | 1.021 |
|  | Late | 2020 | 0.347 | 0.674 | 0.307 | 0.102 | 0.530 | 0.066 | 6.361 | 6.346 | 1.000 |
|  |  | 2021 | 0.360 | 0.674 | 0.324 | 0.115 | 0.541 | 0.074 | 6.432 | 6.344 | 1.000 |
|  |  | 2022 | 0.371 | 0.672 | 0.322 | 0.125 | 0.541 | 0.074 | 6.318 | 6.032 | 1.047 |
| **licensed (assistant) physicians** | Early | 2014 | 0.311 | 0.641 | 0.287 | 0.093 | 0.501 | 0.058 | 6.823 | 6.228 | 1.096 |
|  |  | 2015 | 0.217 | 0.642 | 0.295 | 0.097 | 0.504 | 0.061 | 6.684 | 6.18 | 1.081 |
|  |  | 2016 | 0.305 | 0.650 | 0.295 | 0.082 | 0.495 | 0.060 | 6.66 | 6.332 | 1.052 |
|  | Mid | 2017 | 0.304 | 0.653 | 0.310 | 0.083 | 0.499 | 0.067 | 6.652 | 6.288 | 1.044 |
|  |  | 2018 | 0.333 | 0.668 | 0.330 | 0.096 | 0.531 | 0.076 | 6.759 | 6.278 | 1.077 |
|  |  | 2019 | 0.326 | 0.663 | 0.301 | 0.090 | 0.518 | 0.063 | 6.464 | 6.259 | 1.033 |
|  | Late | 2020 | 0.326 | 0.655 | 0.285 | 0.091 | 0.500 | 0.056 | 6.349 | 6.346 | 1.000 |
|  |  | 2021 | 0.343 | 0.657 | 0.303 | 0.105 | 0.512 | 0.064 | 6.436 | 6.344 | 1.001 |
|  |  | 2022 | 0.353 | 0.656 | 0.303 | 0.114 | 0.514 | 0.065 | 6.303 | 6.302 | 1.000 |
| **Registered nurses** | Early | 2014 | 0.349 | 0.677 | 0.320 | 0.116 | 0.565 | 0.071 | 7.551 | 6.228 | 1.212 |
|  |  | 2015 | 0.354 | 0.677 | 0.325 | 0.119 | 0.566 | 0.074 | 7.326 | 6.18 | 1.185 |
|  |  | 2016 | 0.344 | 0.687 | 0.325 | 0.102 | 0.554 | 0.073 | 7.41 | 6.332 | 1.17 |
|  | Mid | 2017 | 0.344 | 0.689 | 0.342 | 0.101 | 0.555 | 0.082 | 7.311 | 6.288 | 1.163 |
|  |  | 2018 | 0.378 | 0.710 | 0.363 | 0.119 | 0.598 | 0.093 | 7.497 | 6.278 | 1.194 |
|  |  | 2019 | 0.367 | 0.703 | 0.337 | 0.111 | 0.579 | 0.080 | 7.19 | 6.259 | 1.149 |
|  | Late | 2020 | 0.371 | 0.696 | 0.324 | 0.115 | 0.566 | 0.074 | 7.109 | 6.346 | 1.12 |
|  |  | 2021 | 0.383 | 0.695 | 0.342 | 0.128 | 0.574 | 0.083 | 7.079 | 6.344 | 1.121 |
|  |  | 2022 | 0.394 | 0.691 | 0.339 | 0.139 | 0.574 | 0.082 | 6.871 | 6.302 | 1.09 |
| *Prefecture-level city GDP (billion yuan), permanent population (ten thousand people), land area (km²), and health personnel classification data are sourced from the health statistics data of the Zhejiang Health Commission and the 2015-2023 Statistical Yearbooks of Yangtze River Delta. | | | | | | | | | | | |

| Supplementary table 22 The Gini coefficient, Theil index, and **Health Resource Agglomeration Degree** of three categories of health human resources in Anhui Province from 2014 to 2022. | | | | | | | | | | | |
| --- | --- | --- | --- | --- | --- | --- | --- | --- | --- | --- | --- |
| **health workers** | **Year** | | **Gini coefficients** | | | **Theil Index** | | | **Health Resource Agglomeration Degree** | | |
|  |  |  | **Population-based allocation** | **Geographic area-based allocation** | **GDP-based allocation** | **Population-based allocation列** | **Geographic area-based allocation** | **GDP-based allocation** | **HRAD** | **PAD** | **HRAD/PAD** |
| **Health technicians** | Early | 2014 | 0.133 | 0.281 | 0.187 | 0.012 | 0.054 | 0.025 | 0.491 | 0.729 | 0.674 |
|  |  | 2015 | 0.115 | 0.283 | 0.205 | 0.009 | 0.054 | 0.030 | 0.487 | 0.734 | 0.664 |
|  |  | 2016 | 0.114 | 0.303 | 0.200 | 0.009 | 0.063 | 0.029 | 0.484 | 0.734 | 0.660 |
|  | Mid | 2017 | 0.118 | 0.305 | 0.198 | 0.010 | 0.064 | 0.028 | 0.483 | 0.736 | 0.656 |
|  |  | 2018 | 0.113 | 0.311 | 0.206 | 0.009 | 0.067 | 0.031 | 0.483 | 0.741 | 0.651 |
|  |  | 2019 | 0.120 | 0.319 | 0.163 | 0.011 | 0.071 | 0.018 | 0.490 | 0.743 | 0.660 |
|  | Late | 2020 | 0.071 | 0.309 | 0.188 | 0.003 | 0.065 | 0.024 | 0.524 | 0.705 | 0.744 |
|  |  | 2021 | 0.067 | 0.310 | 0.195 | 0.003 | 0.066 | 0.026 | 0.529 | 0.71 | 0.744 |
|  |  | 2022 | 0.053 | 0.309 | 0.204 | 0.002 | 0.066 | 0.029 | 0.547 | 0.706 | 0.775 |
| **licensed (assistant) physicians** | Early | 2014 | 0.122 | 0.269 | 0.198 | 0.011 | 0.049 | 0.028 | 0.542 | 0.729 | 0.744 |
|  |  | 2015 | 0.114 | 0.268 | 0.213 | 0.009 | 0.049 | 0.032 | 0.534 | 0.734 | 0.728 |
|  |  | 2016 | 0.107 | 0.288 | 0.208 | 0.008 | 0.057 | 0.031 | 0.523 | 0.734 | 0.713 |
|  | Mid | 2017 | 0.108 | 0.294 | 0.206 | 0.008 | 0.059 | 0.031 | 0.526 | 0.736 | 0.714 |
|  |  | 2018 | 0.104 | 0.301 | 0.209 | 0.008 | 0.062 | 0.032 | 0.517 | 0.741 | 0.698 |
|  |  | 2019 | 0.111 | 0.309 | 0.166 | 0.010 | 0.066 | 0.019 | 0.521 | 0.734 | 0.701 |
|  | Late | 2020 | 0.058 | 0.300 | 0.195 | 0.002 | 0.062 | 0.026 | 0.574 | 0.705 | 0.814 |
|  |  | 2021 | 0.055 | 0.303 | 0.201 | 0.002 | 0.064 | 0.028 | 0.578 | 0.705 | 0.814 |
|  |  | 2022 | 0.052 | 0.302 | 0.207 | 0.002 | 0.062 | 0.030 | 0.589 | 0.706 | 0.834 |
| **Registered nurses** | Early | 2014 | 0.181 | 0.309 | 0.156 | 0.022 | 0.066 | 0.017 | 0.552 | 0.729 | 0.758 |
|  |  | 2015 | 0.150 | 0.306 | 0.172 | 0.015 | 0.065 | 0.021 | 0.547 | 0.734 | 0.746 |
|  |  | 2016 | 0.145 | 0.328 | 0.183 | 0.014 | 0.075 | 0.025 | 0.543 | 0.734 | 0.74 |
|  | Mid | 2017 | 0.150 | 0.329 | 0.180 | 0.015 | 0.076 | 0.023 | 0.544 | 0.736 | 0.739 |
|  |  | 2018 | 0.141 | 0.332 | 0.193 | 0.014 | 0.077 | 0.027 | 0.542 | 0.741 | 0.731 |
|  |  | 2019 | 0.149 | 0.337 | 0.146 | 0.017 | 0.080 | 0.015 | 0.547 | 0.743 | 0.737 |
|  | Late | 2020 | 0.093 | 0.323 | 0.179 | 0.006 | 0.071 | 0.022 | 0.588 | 0.705 | 0.834 |
|  |  | 2021 | 0.084 | 0.322 | 0.187 | 0.005 | 0.071 | 0.024 | 0.592 | 0.705 | 0.834 |
|  |  | 2022 | 0.070 | 0.322 | 0.195 | 0.003 | 0.071 | 0.026 | 0.617 | 0.706 | 0.874 |
| *Prefecture-level city GDP (billion yuan), permanent population (ten thousand people), land area (km²), and health personnel classification data are sourced from the health statistics data of the Zhejiang Health Commission and the 2015-2023 Statistical Yearbooks of Yangtze River Delta. | | | | | | | | | | | |

Supplementary table 23 Trend Analysis of Fairness in Health Human Resource Allocation Based on the Gini Coefficient.

|  | **Population-based allocation** | | | **Geographic area-based allocation** | | | **GDP-based allocation** | | |
| --- | --- | --- | --- | --- | --- | --- | --- | --- | --- |
|  | **Health technicians** | **licensed (assistant) physicians** | **Registered nurses** | **Health technicians** | **licensed (assistant) physicians** | **Registered nurses** | **Health technicians** | **licensed (assistant) physicians** | **Registered nurses** |
| Zhejiang | 0.0005(-0.0004,0.0013) | 0.0015(0.0007，0.0022)** | -0.0007(-0.0019,0.0006) | 0.0021(0.0009,0.0033)* | 0.0041(0.0024,0.0058)** | 0.0012(-0.0001,0.0026) | -0.0036(-0.0078,0.0006) | -0.0009(-0.0027,0.0008) | 0.0013(0.0000,0.0026) |
| Jiangsu | 0.0023(-0.0004,0.0050) | 0.0005(-0.0016,0.0027) | -0.0016(-0.0043,0.0011) | 0.0082(0.0074,0.0090)*** | 0.0063(0.0049,0.0076)** | 0.0040(0.0031,0.0050)*** | -0.0061(-0.0070,-0.0051)*** | -0.0027(-0.0035,-0.0018)*** | -0.0018(-0.0031,-0.0005)* |
| Shanghai | 0.005(0.0025,0.0076)* | 0.0102(0.0025,0.0178)* | 0.0057(0.0033,0.0082)** | 0.0020(0.0000 0.0038) | 0.0021(0.0000,0.0039) | 0.0024(-0.0000,0.0047) | 0.0015(-0.0017,0.0047) | 0.00098(-0.0026,0.0046) | 0.002(-0.0013,0.0053) |
| Anhui | -0.0091(-0.0129,-0.0054)** | -0.0092(-0.0128,-0.0056)** | -0.0125(-0.0166,-0.0083)*** | 0.0037(0.0015,0.0058)* | 0.0046(0.0024,0.0068)** | 0.0016(-0.0008,0.0041) | -0.0004(-0.0040,0.0033) | -0.0011(-0.0048,0.0026) | 0.0027(-0.0013,0.0066) |

*p.value < 0.001* ~ "***", *p.value < 0.01* ~ "**",  *p.value < 0.05* ~ "*".

Supplementary table 24 Trend Analysis of Fairness in Health Human Resource Allocation Based on the Thiel Index.

|  | **Population-based allocation** | | | **Geographic area-based allocation** | | | **GDP-based allocation** | | |
| --- | --- | --- | --- | --- | --- | --- | --- | --- | --- |
|  | **Health technicians** | **licensed (assistant) physicians** | **Registered nurses** | **Health technicians** | **licensed (assistant) physicians** | **Registered nurses** | **Health technicians** | **licensed (assistant) physicians** | **Registered nurses** |
| Yangtze River Delta | -0.0007(-0.0013,-0.0001) | -0.0004(-0.0010,0.0001) | -0.0008(-0.0012,-0.0003)* | 0.0008(-0.0005,0.0022) | -0.0003(-0.0016,0.0011) | -0.0015(-0.00311,0.0000) | -0.0006(-0.0010,-0.0001)* | -0.0003(-0.0009,0.0003) | 0.0002(-0.0005,0.0009) |
| Zhejiang | -0.0001(-0.0002,0.0000 | 0.0000(-0.0000,,0.0002) | -0.0003(-0.0005,-0.0001)* | 0.0008(0.0003,0.0013)* | 0.0014(0.0008,0.0019)** | 0.0006(0.0001,0.0011)* | 0.0001(0.0002,0.0003) | -0.0002(-0.0005,0.0001) | 0.0002(0.0000,0.0004) |
| Jiangsu | 0.0003(0.0000,0.0006) | 0.0001(0.0000,0.0003) | -0.0001(-0.0005,0.0002) | 0.0034(0.0032,0.0037)*** | 0.0025(0.0020,0.0031)** | 0.0017(0.0013,0.0020)*** | -0.0003(-0.0013,0.0008) | -0.00053(-0.0008,-0.0002)** | -0.0002(-0.0005,0.0000) |
| Shanghai | 0.0021(-0.0004,0.0045) | 0.0022(0.0000,0.0044) | 0.0026(-0.0000,0.0052) | 0.0011(-0.0018,0.0041) | 0.0017(-0.0011,0.0046) | 0.0018(-0.0016,0.0052) | 0.0008(-0.000,0.0024) | 0.0004(-0.0011,0.0020) | 0.0012(-0.0005,0.0028) |
| Anhui | -0.0012(-0.0017,-0.0006)** | -0.0011(-0.0017,-0.0006)** | -0.0020(-0.0028,-0.0012)** | 0.0016(0.0006,0.0026)* | 0.0019(0.0010,0.0028)** | 0.0006(-0.0007,0.0019) | -0.0003(-0.0013,0.0008) | -0.0004(-0.0015,0.0006) | 0.0005(-0.0005,0.0015) |

*p.value < 0.001* ~ "***", *p.value < 0.01* ~ "**",  *p.value < 0.05* ~ "*".

Supplementary table 25 Trend Analysis of Fairness in Health Human Resource Allocation Based on Health Resource Agglomeration Degree.

|  | **HRAD** | | | **HRAD/PAD** | | |
| --- | --- | --- | --- | --- | --- | --- |
|  | **Health technicians** | **licensed (assistant) physicians** | **Registered nurses** | **Health technicians** | **licensed (assistant) physicians** | **Registered nurses** |
| Zhejiang | 0.0000(-0.0025,0.0025) | 0.0004(-0.0030,0.0038） | 0.0005(-0.0024,0.0034) | -0.0003(-0.0037,0.0031) | -0.0008(-0.0057,0.0041) | 0.0000(0.0000,0.0039) |
| Jiangsu | -0.0101(-0.0140,-0.0062)** | -0.0063(-0.0130,0.0004) | -0.0074(-0.0121,-0.0027)* | \| -0.01086(-0.0167,-0.0050) \| \| --- \|   ** | -0.0068(-0.0143,0.0007) | -0.0084(-0.0145,-0.0023)* |
| Shanghai | 0.0027(-0.0228,0.0282) | -0.0606(-0.0834,-0.0378)** | -0.0697(-0.0985,-0.0410)** | 0.0005(-0.0045,0.0054) | -0.0123(-0.0165,-0.0081)*** | -0.0132(-0.0176,-0.0089)*** |
| Anhui | 0.0073(0.0035,0.0111)** | 0.0069(0.0015,0.0124)* | 0.0081(0.0036,0.0127)** | 0.0136(0.0054,0.0218)* | 0.0135(0.0027,0.02422)* | 0.0152(0.0057,0.0247)* |

*p.value < 0.001* ~ "***", *p.value < 0.01* ~ "**",  *p.value < 0.05* ~ "*".

Supplementary table 26 Trend Analysis of Fairness in Health Human Resource Allocation Based on the Gini Coefficient.

| **Province** | **Population-based allocation** | | | **Geographic area-based allocation** | | | **GDP-based allocation** | | |
| --- | --- | --- | --- | --- | --- | --- | --- | --- | --- |
|  | **Health technicians** | **licensed (assistant) physicians** | **Registered nurses** | **Health technicians** | **licensed (assistant) physicians** | **Registered nurses** | **Health technicians** | **licensed (assistant) physicians** | **Registered nurses** |
| Zhejiang | 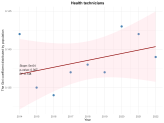 | 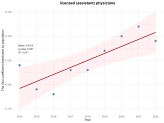 | 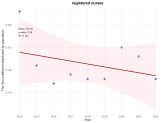 | 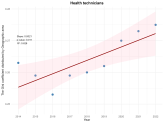 | 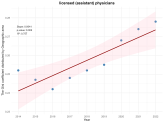 | 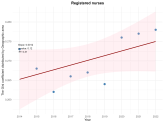 | 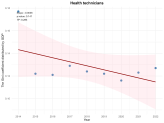 | 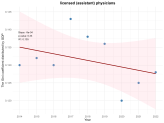 | 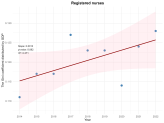 |
| Jiangsu | 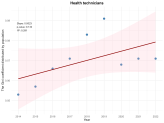 | 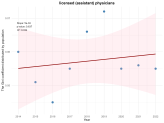 | 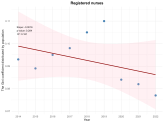 | 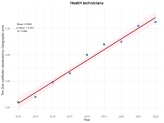 | 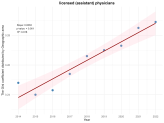 | 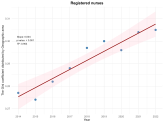 | 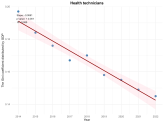 | 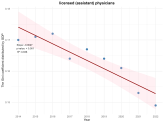 | 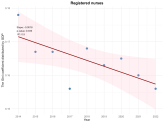 |
| Shanghai | 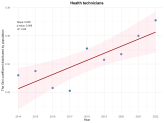 | 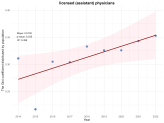 | 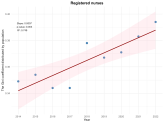 | 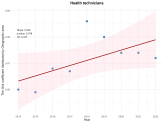 | 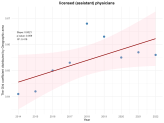 | 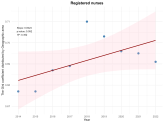 | 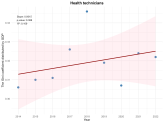 | 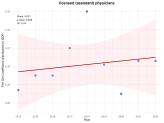 | 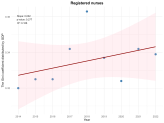 |
| Anhui | 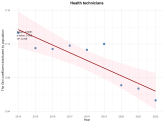 | 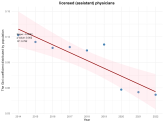 | 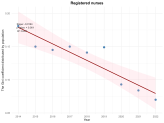 | 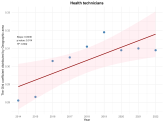 | 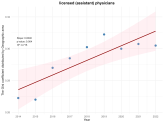 | 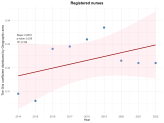 | 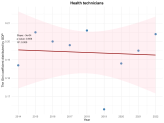 | 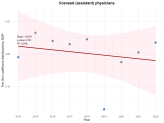 | 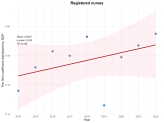 |

Supplementary table 27 Trend Analysis of Fairness in Health Human Resource Allocation Based on the Thiel Index.

| **Province** | **Population-based allocation** | | | **Geographic area-based allocation** | | | **GDP-based allocation** | | |
| --- | --- | --- | --- | --- | --- | --- | --- | --- | --- |
|  | **Health technicians** | **licensed (assistant) physicians** | **Registered nurses** | **Health technicians** | **licensed (assistant) physicians** | **Registered nurses** | **Health technicians** | **licensed (assistant) physicians** | **Registered nurses** |
| Yangtze River Delta | 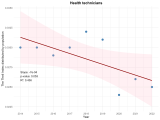 | 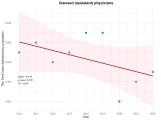 | 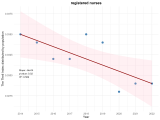 | 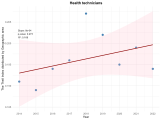 | 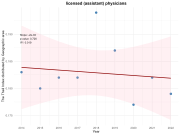 | 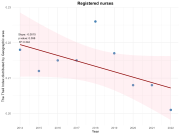 | 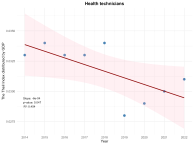 | 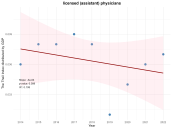 | 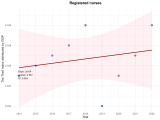 |
| Zhejiang | 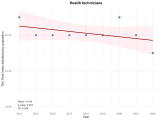 | 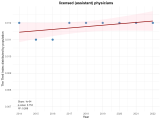 | 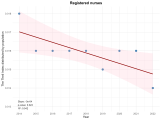 | 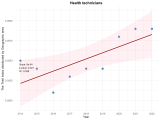 | 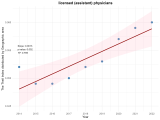 | 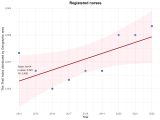 | 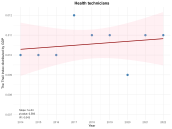 | 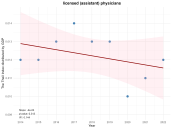 | 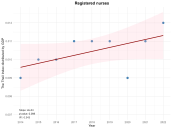 |
| Jiangsu | 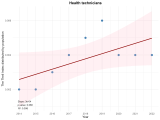 | 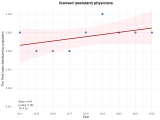 | 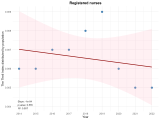 | 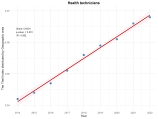 | 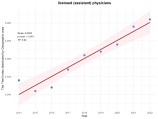 | 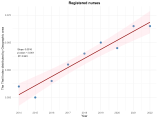 | 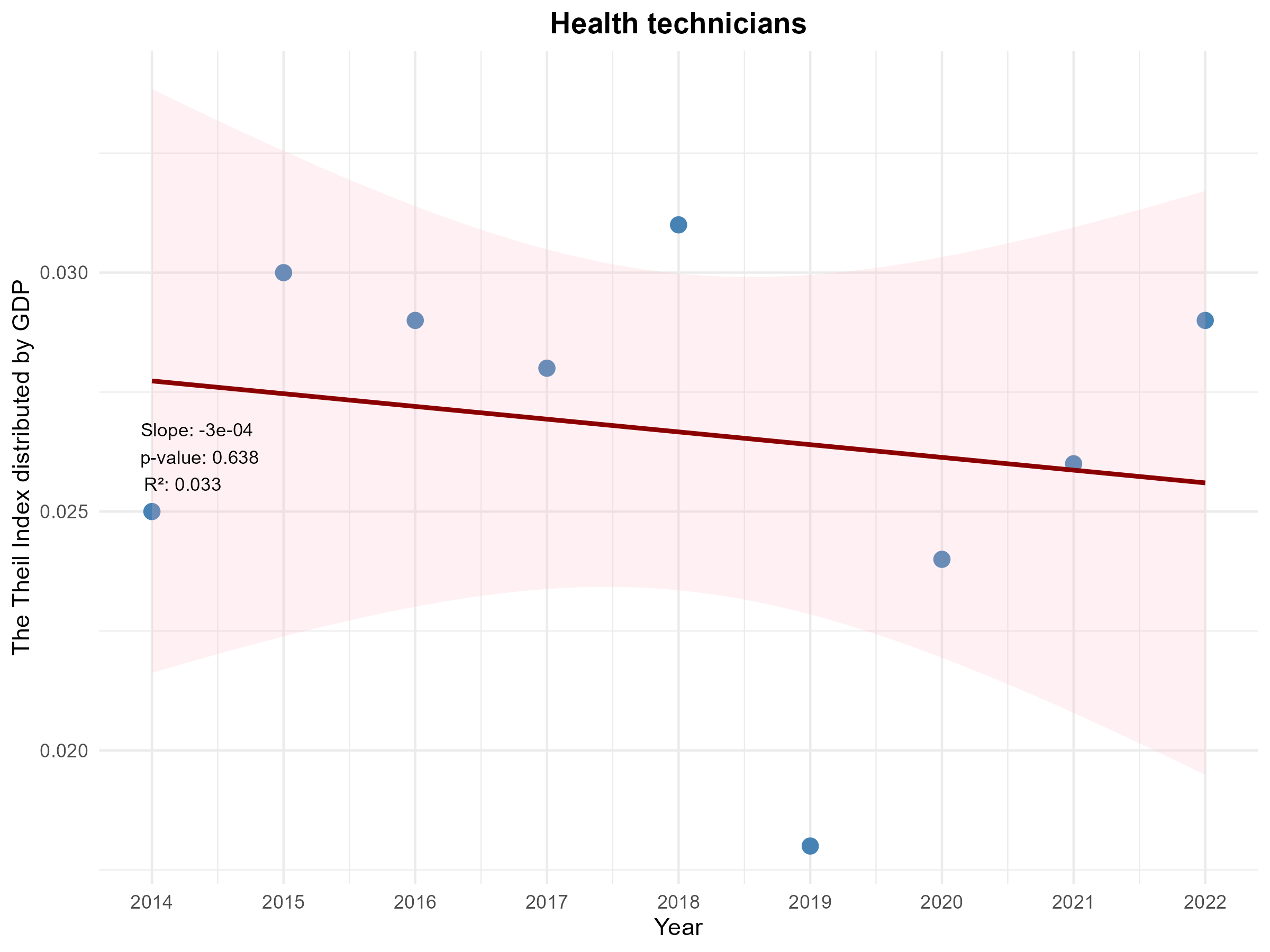 | 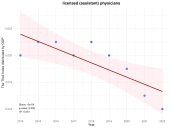 | 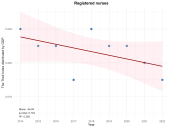 |
| Shanghai | 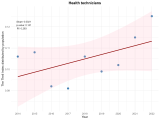 | 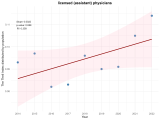 | 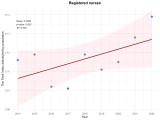 | 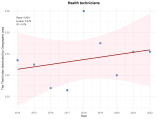 | 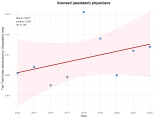 | 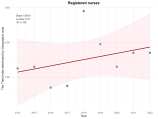 | 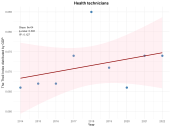 | 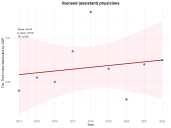 | 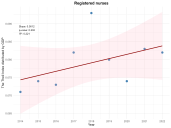 |
| Anhui | 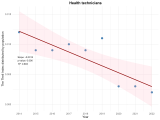 | 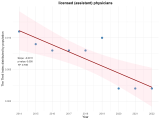 | 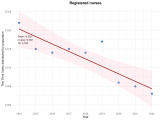 | 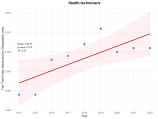 | 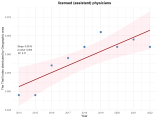 | 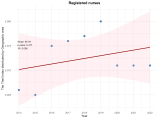 | 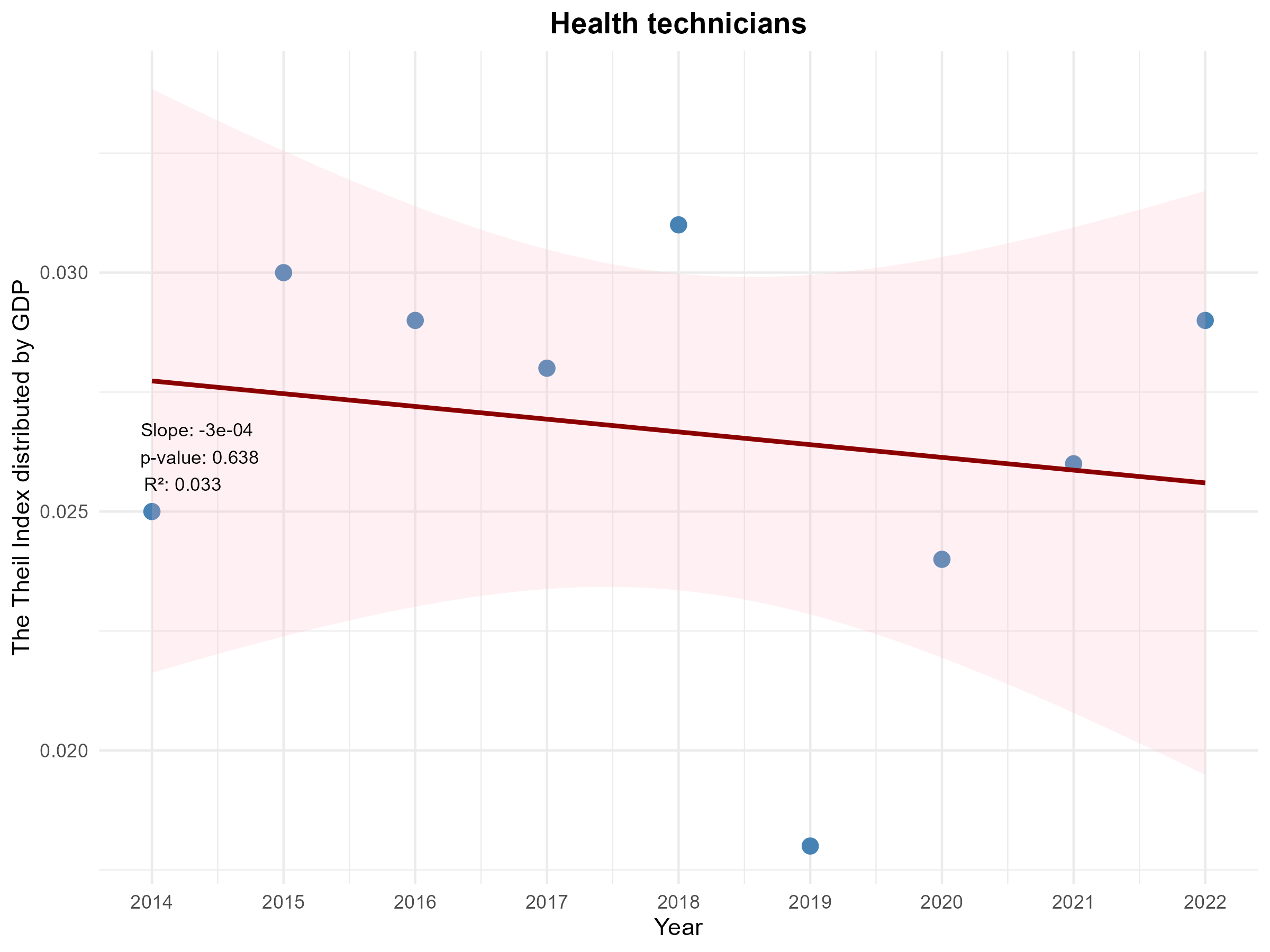 | 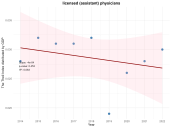 | 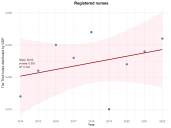 |

Supplementary table 28 Trend Analysis of Fairness in Health Human Resource Allocation Based on Health Resource Agglomeration Degree.

| **Province** | HRAD | | | HRAD/PAD | | |
| --- | --- | --- | --- | --- | --- | --- |
|  | **Health technicians** | **licensed (assistant) physicians** | **Registered nurses** | **Health technicians** | **licensed (assistant) physicians** | **Registered nurses** |
| Zhejiang | 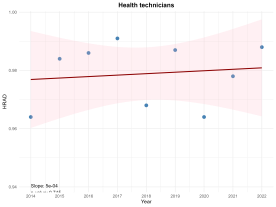 | 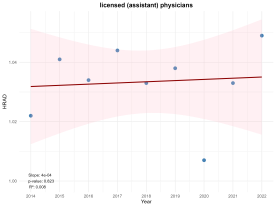 | 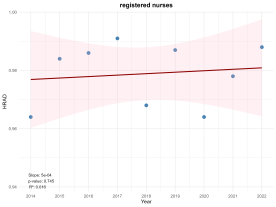 | 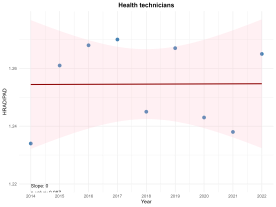 | 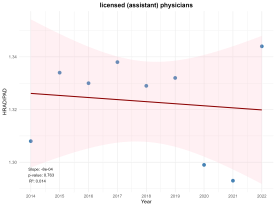 | 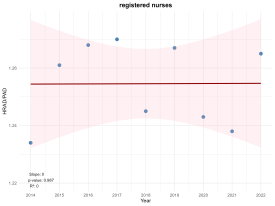 |
| Jiangsu | 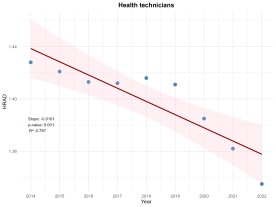 | 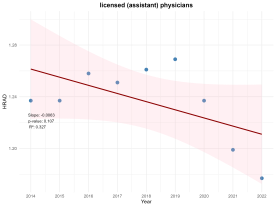 | 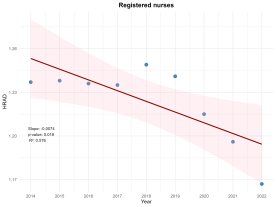 | 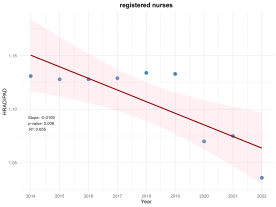 | 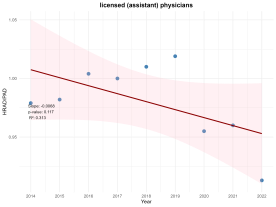 | 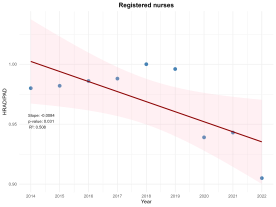 |
| Shanghai | 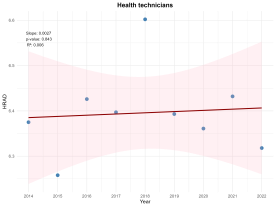 | 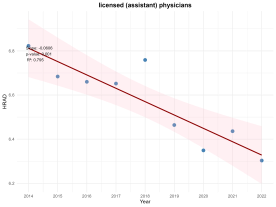 | 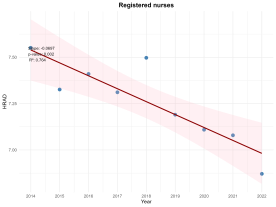 | 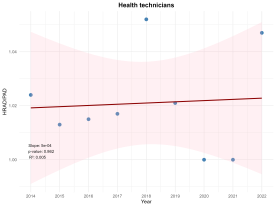 | 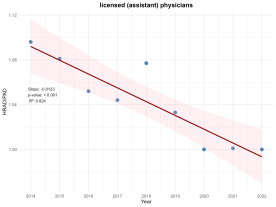 | 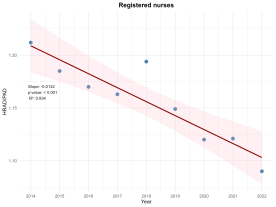 |
| Anhui | 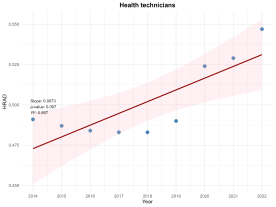 |  |  |  |  |  |
